# Genolator enables protein function interpretation using a multimodal large language model fusing genomic and structural interpretation with natural language interaction

**DOI:** 10.1186/s13059-026-04274-w

**Published:** 2026-09-16

**Authors:** Martin Danner, Tanhim Islam, Matthias Begemann, Florian Kraft, Miriam Elbracht, Ingo Kurth, Jeremias Krause

**Affiliations:** 1 Center for Human Genetics and Genomic Medicine, Medical Faculty, Uniklinik RWTH Aachen, Pauwelsstrasse 30, D-52074 Aachen, Germany; 2 scieneers GmbH, Karlsruhe, Germany

**Keywords:** Genomic language models, Multimodal language models, Protein function prediction, Feature fusion, Genolator

## Abstract

**Background:**

Decoding the genetic code to unveil its genome functionality is a monumental task which would greatly advance the understanding of disease mechanisms and development of targeted treatments. Although large language models (LLMs) have transformed natural language processing across diverse domains, translating the complex language of DNA into human-readable form remains challenging due to genomic data complexity and unexplored regions of the human genome. Current language models either are capable of processing natural language or the genomic code. Models fusing both aspects are largely lacking.

**Results:**

Here we present Genolator, a multimodal large language model that integrates embeddings from DNA sequences, amino acid sequences, and protein structures with natural language queries. Fine-tuned on over 365,000 question–answer pairs generated using abstracted Gene-Ontology (GO) terms, Genolator effectively answers queries regarding protein subcellular localization, molecular function, and biological processes. Evaluation demonstrates high accuracy in confirming or denying protein function associations, outperforming baseline models such as openly available allrounder LLMs like GPT 4.1 as well as smaller domain-specific models integrating knowledge from a protein structure transformer. Explorations of Genolator’s hidden states unveil a biologically and linguistically plausible organization of its learned representations. Analysis of the attention heads of the underlying language model and an ablation study provide evidence for a benefit of the multi-modal approach.

**Conclusion:**

Genolator enhances accessibility to genomic information by enabling natural language interaction with protein data, facilitating biological discovery, and clinical research. It represents a step towards bridging genomic code and human language through the integration of a multimodal LLM.

**Supplementary Information:**

The online version contains supplementary material available at https://doi.org/10.1186/s13059-026-04274-w.

## Background

Proteins and nucleic acids, alongside lipids and glycans, are fundamental molecules in cells [1, 2]. They enable biological functions, store genetic information, and serve as essential building blocks of the cell. The human genome and proteome are vastly complex, and to this day a large portion of the life’s code remains unexplored [2–5]. Yet, deciphering the molecular function of proteins and their involvement in biological processes is key to understanding disease mechanisms and designing targeted treatments [6–8].

While experimental characterization remains the gold standard for determining protein function, it is inherently resource- and time-intensive, limiting its scalability. To address these constraints, numerous computational methods have emerged to reduce experimental workload and broaden our understanding of the human genome. By prioritizing likely functional candidates for follow-up, these approaches can significantly accelerate downstream experimental validation [7–16]. Among computational approaches, machine learning models are increasingly employed for predicting the functionality and cellular localization of proteins [15]. These machine learning models typically treat protein function prediction as a multi-label problem task in which associated functionalities (encoded as GO-terms) are predicted. To achieve this, these models leverage different modalities, including protein-structure information and sequence information. Part of the utilized machine learning models are large language models (LLMs) which recently demonstrated significant impact across virtually all domains of life [17], with applications spanning medicine [18], education [19], traffic management [20], finance [21] and beyond. Leveraging billions of parameters, these machine learning models can discern intricate patterns in data, enabling them to understand and generate human language and solve a diverse range of complex natural language processing tasks, making them prime candidates to decode the information stored in the genetic code. Therefore, genomic language models have been developed which extend the concept of large language models to genomic language. By adopting LLM-like architectures and incorporating specific adaptations, as in models such as Evo2 [22, 23], genomic language models are trained on trillions of DNA base pairs from a wide range of prokaryotic and eukaryotic species [22–27]. This allows them to interpret and understand genetic information, predict the functional impacts of genetic variation, and identify biological features such as exon–intron boundaries, binding sites, and even aspects of protein functionality and structure [22, 23]. The knowledge acquired during training is encoded within the model’s parameters, which serve as a foundation for various downstream tasks. However, current genomic language models are mostly inaccessible to non-technical users, need to be finetuned on each downstream task and most importantly lack an understanding of natural language which could prove crucial to decoding the genomic code more easily [28]. Finally, current genomic language models are typically limited to specific input modalities (DNA, amino acid sequences or protein structure information), highlighting the need for explorations of unifying their specialist predictions using multi-modal model architectures. Formulating questions about genomic aspects such as gene functionality in natural language and thereby understanding how the information about the gene functionality is encoded in the genetic material as well as combining predictions from modality-specific approaches using a unifying multi-modal model, could open a new way to explore and decipher genome biology.

Building on this conceptual foundation and the aforementioned challenges, our study introduces Genolator: an accessible model capable of processing genomic information and natural language in tandem, facilitating the exploration of the human genome through natural language queries. For a proof of concept, we selected the decoding of the functionality of protein coding genes and subsequently proteins as our prime task. Our focus is to enable users to ask and obtain answers to questions about the biological process, molecular function, and cellular component of a given (potentially novel) protein, as derived from the Gene Ontology (GO) nomenclature [29], a standardized framework for the description of protein functionality. In previous work [30], we demonstrated that sequence embeddings capture significant information but may not encompass all relevant biological features needed for various downstream tasks. To address this, we adopt an intermediate fusion strategy [31, 32], combining four distinct modalities: the DNA sequence, the amino acid sequence, the 3D protein structure of the target gene or protein, and the user’s natural language query.

## Results

### Genolator – a multimodal large language model regarding gene functionality

Genolator is a finetuned multimodal Llama [33–35] model, designed to fuse natural language with genomic language. It receives a question, formulated in natural language (English), and information from DNA-sequences, amino acid sequences and protein structures in tandem. We experimented using two versions of the Genolator:

1. A Multimodal model fusing DNA-Sequences with Amino Acid Sequences
2. A Multimodal model fusing DNA-Sequencies with structural enriched protein sequences

Each modality is presented to Genolator in the form of an embedding, a latent representation of the encoded information, from a machine learning model trained on the specific modality. DNA sequences are handled by the genomic foundation model Evo2 [22], amino acids by the protein language model ESM2 [36] and protein structure-infused amino acid embeddings created by PST [37]. Genolator utilizes token projectors and in batch token scaling to fuse the input from the modality encoders with the natural language question and presents the output to a Llama model which processes the input and formulates a response which will be returned in English. Genolator was designed to answer questions regarding gene functionality. To achieve this, gene functionality descriptions were derived from the three GO-Term [29] aspects:

1. Cellular component
2. Molecular function
3. Biological processes

**Fig. 1:**
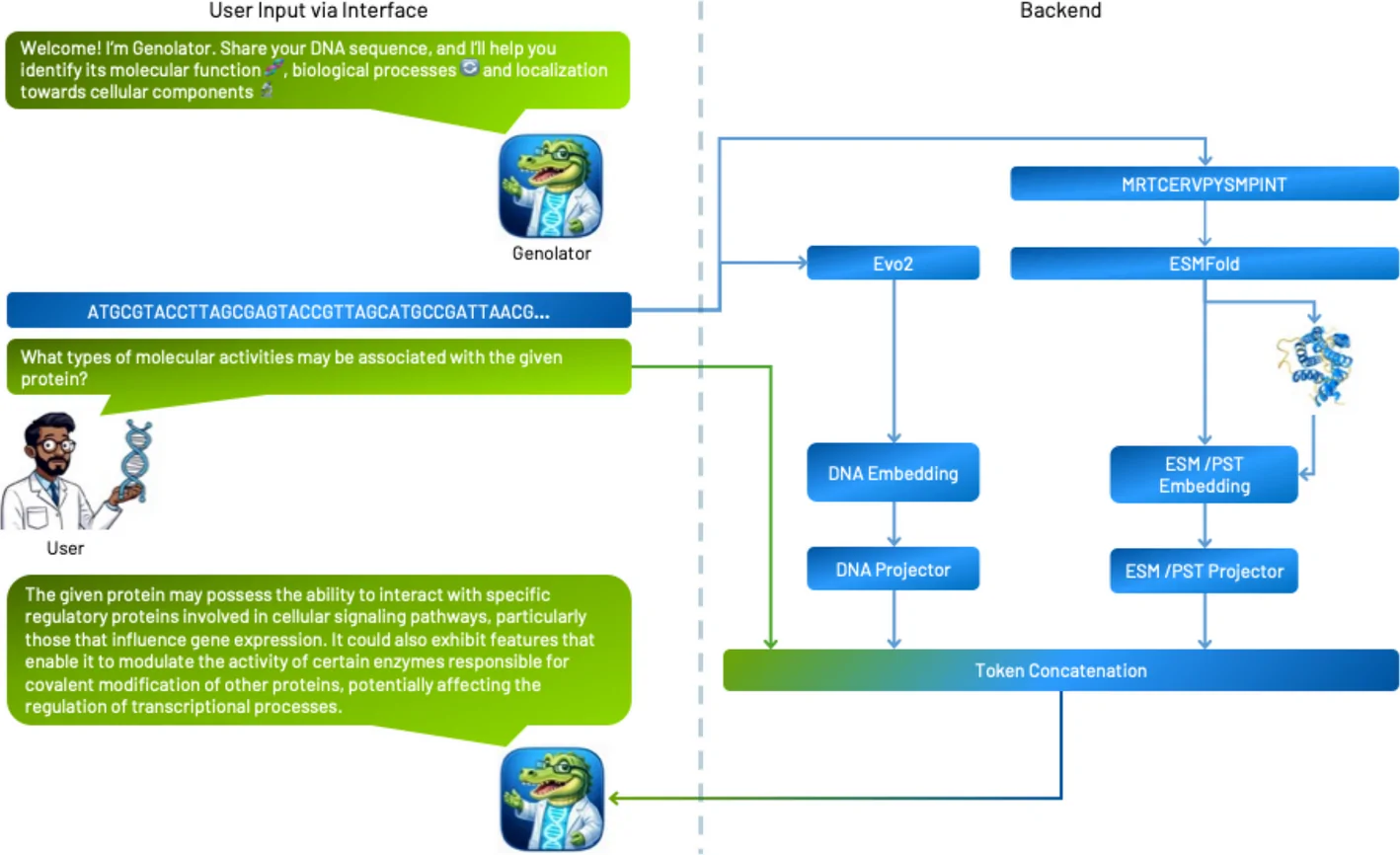
Representative illustration of a user assessing the molecular function of a DNA sequence with a generic query. Left: The conversation between Genolator and the user. Right: A schematic overview of the backend process handling the user’s query and parsing of the provided DNA sequence

**Fig. 2:**
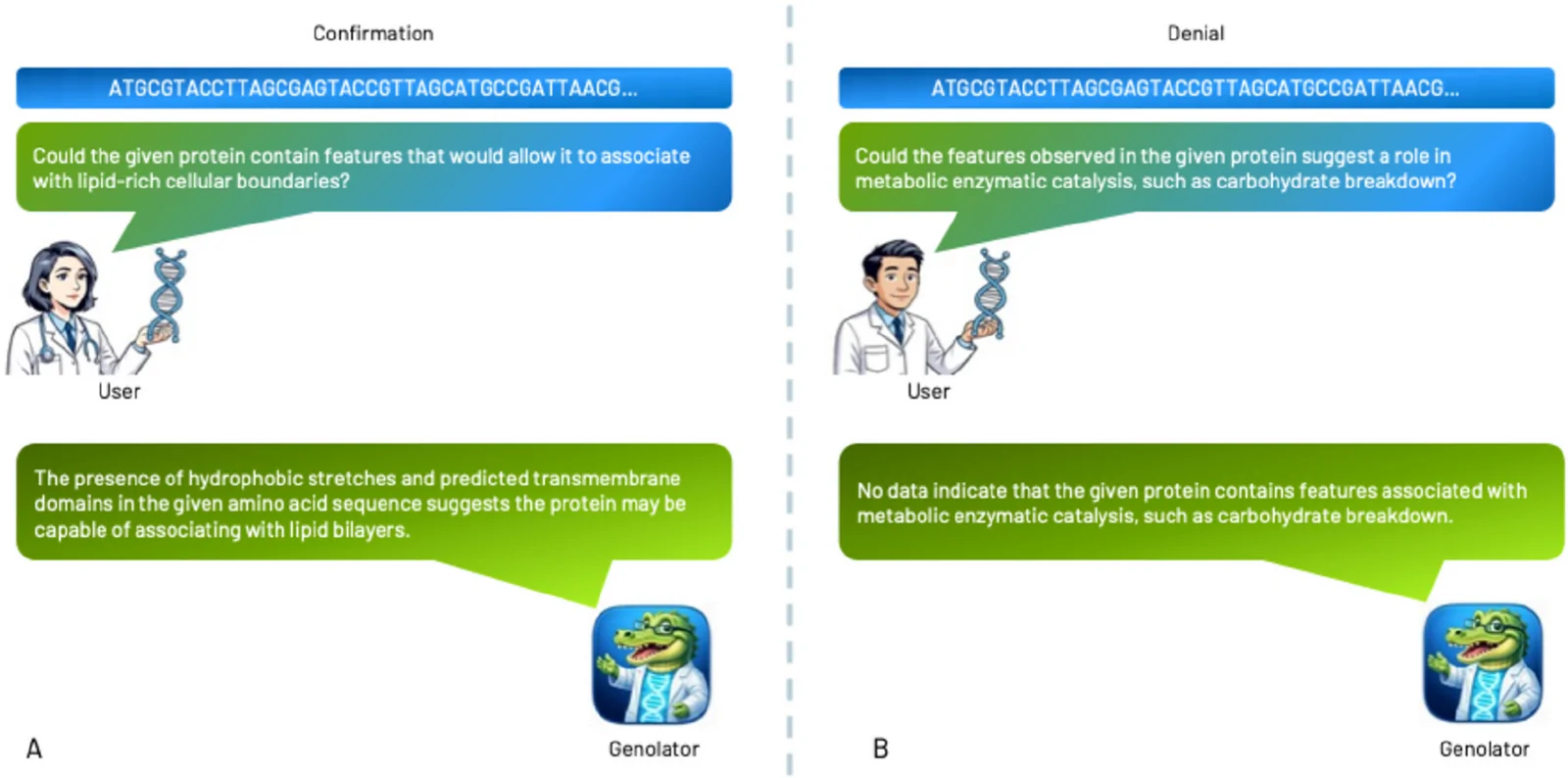
**A** Illustration of a sample user interaction with Genolator, in which the user queries whether a sequence of interest is associated with a specific cellular component, and Genolator confirms the association. **B** Schematic illustration of a user querying Genolator about the involvement of the given sequence in a particular biological process, which is denied by Genolator

Which were transformed into a generally understandable phrase by a GPT 4.1 model [38]. Genolator was subsequently trained on over 365,000 question–answer pairs encompassing both genuine protein function associations and denial questions, where incorrect associations were deliberately constructed. The training set included both confirmative and denial questions, which required responses affirming or denying specific associations, as well as open-ended questions demanding more complex, explanatory answers. As a result, Genolator can address both open-ended queries (see Fig. 1) and confirmatory or denial questions (see Fig. 2) relating to gene functionality.

### Genolator can accurately confirm and deny gene functionality associations and outperforms allrounder and specialist baseline models

To assess the accuracy and validity of Genolator, we first evaluated its performance on a holdout test set comprising 34,743 questions. This set included confirmative (*n* = 15,463) and denial (*n* = 14,943) questions addressing subcellular localization, molecular function, and involvement in biological processes, specifically for proteins and genes that were entirely unseen during training. Additionally, to evaluate Genolator on proteins with unknown function, we employed a custom similarity-aware KMeans-based [39] data split. This splitting strategy ensured that test set samples were not only unseen during training but also resided in distinct regions of the joint multimodal embedding space, thus minimizing structural or sequence similarity to the training data across all modalities. To evaluate Genolator’s performance on the task to answer denial and confirmative questions the task was reframed as a binary classification problem. The performance was evaluated using Accuracy, Precision, Recall, F1-Score and the Matthews-Correlation-Coefficient (MCC) [40]. Genolator (full; Evo2 & PST), in which both the virtual token projectors and the Llama model were jointly trained/fine-tuned, achieved an overall F1-score of around 0.98. For both the confirmation and denial of associated terms, it reached a MCC of 0.959, the full results are displayed in Table 1. The other full model (Evo2 & ESM2) achieved an overall F1-score of 0.97 and a MCC of 0.948. For further quantification of Genolator’s performance, multiple baseline models were benchmarked on the test set. These included general-purpose "all-rounder" LLMs such as GPT 4.1, locally deployable models like the 8B version of Llama-3 [33], and a smaller, domain-specific XGBoost [41] classifier trained on information from the PST model together with BioBERT [42] sentence-transformer embeddings of the input questions. Additionally, we evaluated ablated versions of Genolator (Evo2 + PST/TP-Only Fine-Tuning) and Genolator (Evo2 + ESM2/TP-Only Fine-Tuning), in which only the virtual token projectors were trained while the Llama [33] backbone remained frozen. For GPT 4.1 [38] and the non-finetuned Llama-3 [33] the DNA sequence and the amino acid sequence was directly embedded into the prompt. Both full versions of Genolator outperformed all baseline models, the overall best model was the full model trained on Evo2 and PST embeddings. GPT 4.1 [38] reached a MCC of 0.179 and the XGBoost model reached a MCC of 0.913. The ablated version of Genolator (Evo2 + ESM2/TP-Only Fine-Tuning) reached a MCC of 0.895 and Genolator (Evo2 + PST/TP-Only Fine-Tuning) a MCC of 0.924. The non finetuned Llama model had to be excluded from the performance comparison. Due to the O(n^2^) complexity of the self-attention mechanism, a non-multimodal Llama [33] model encounters memory limitations and excessive computational time when processing large, unembedded DNA sequences. Even when deployed on a high-memory GPU such as the NVIDIA H100, the model faces significant issues with long input sequences, making practical inference infeasible for these inputs. For cases where the base model was capable of producing an answer, the answer highlighted further difficulties of the base model, these being:

1. False remembrance
2. Amino-acid gibberish
3. Answering the question with a follow up question.

**Table 1:**
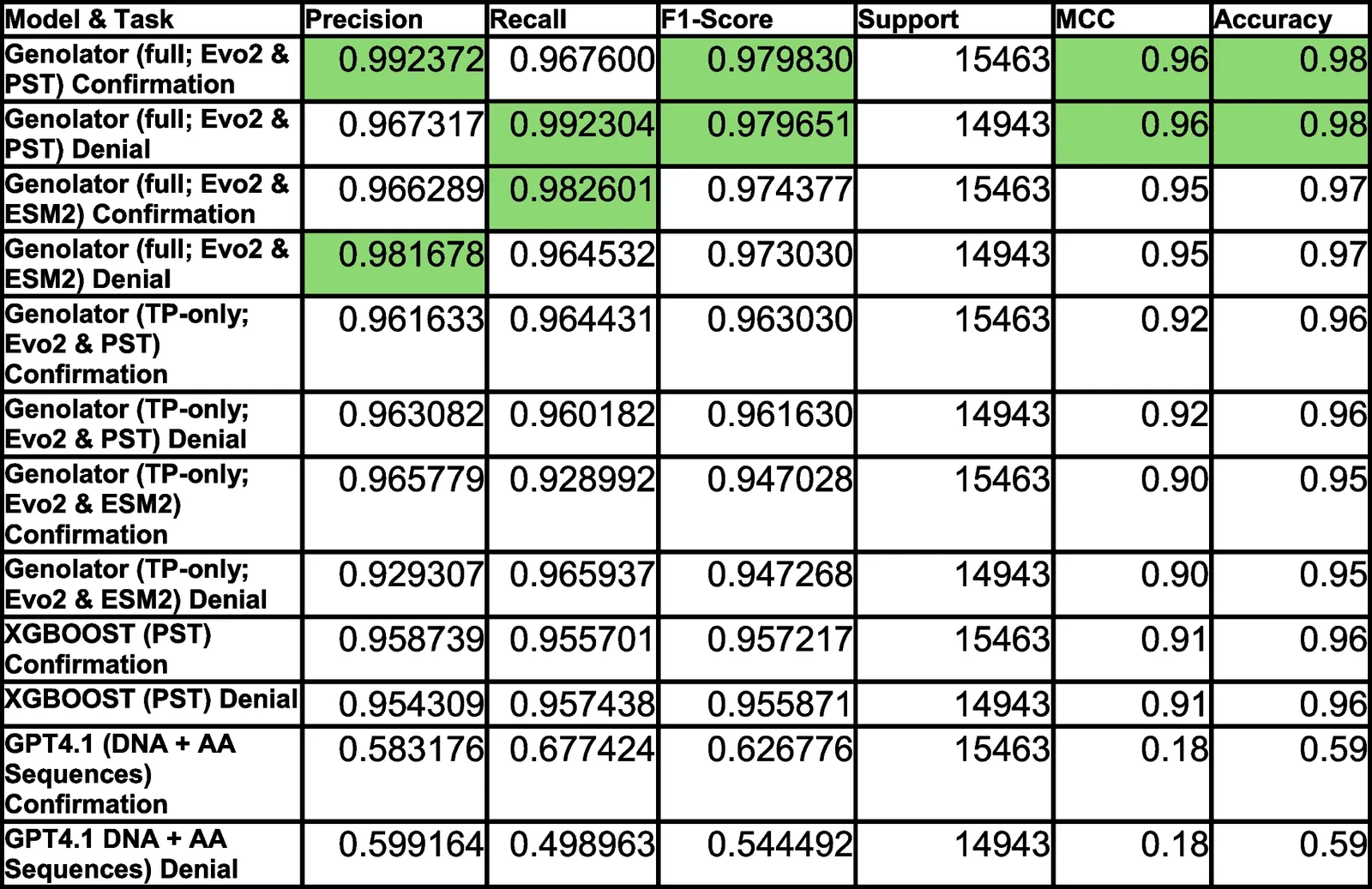
Performance of Genolator (and baseline models) on confirmation/denial questions; green highlights indicate best performance

In case one it seemed like the base model tried to utilize knowledge from its initial training, which was ultimately futile and lead to associations with wrong proteins. In case two the base model ignored the question and returned a generated DNA or Amino Acid Sequence, in a next character-prediction style. In some cases, after generating some characters the model fell into a repeating pattern of amino acids. In case three the base model also ignored the question and returned a (unrelated) question. Examples for answers from the untrained Llama model are attached as an Excel sheet (“Case Study Untrained Llama”) in Additional file 2.

Because predicting gene functionality varies in difficulty [7] across the three Gene Ontology (GO) aspects—molecular function, cellular component, and biological process- we evaluated and compared Genolator's (full) performance with the baseline models for each category. For all models, associating a protein with a molecular function proved to be the easiest task, yielding the highest MCCs. Except for GPT 4.1 [38], which performed better on the biological process aspect, the cellular component task ranked second in difficulty, while predicting involvement in biological processes was the most challenging aspect overall. This is consistent with similar observations made regarding the difficulty across the prediction of GO terms across different aspects [7]. While the ablated projector-only version of Genolator (TP) model outperformed GPT 4.1 [38], the difference of performance compared to the smaller XGBoost [41] model was less obvious. The test set for the confirmation and denial questions can be found in the form of an Excel sheet (“KMeans Split Test Set”) in Additional file 2.

### The non-finetuned publicly available GPT 4.1 model (falsely) remembers genomic data and struggles with the concept of sequence similarity

Publicly available large language models (e.g., ChatGPT) can, in principle, be applied (and probably are applied) to genomic data. However, our previous results show that general-purpose models such as GPT 4.1 [38] and Llama3 [33] (8B parameters) underperform compared to modality-specific approaches like XGBoost [41] or Genolator. To further investigate this limitation, we conducted a focused case study analysing the ability of GPT 4.1 [38] to process nucleotide and amino acid sequences when these are directly embedded within the prompt. For this we presented GPT 4.1 [38] with nucleotide sequences and amino acid sequences of well characterized genes such as *BRCA1* (as a representative for proteins with more than 1000 amino acids), *IHH* (as a representative for proteins with an intermediate length) and *INS*, encoding insulin, (as a representative for small proteins) and asked the model openly formulated questions about the involvement of the corresponding protein in biological processes, the associated molecular functions and the cellular localisation of the protein. Since GPT 4.1 [38] was trained on large volumes of publicly available data, it is possible (and likely) that the model already encountered the genomic information during training. Therefore, in addition to the genomic functionality questions, we also prompted the model to identify the provided nucleotide and amino acid sequence, resulting in three identification queries per case. The summarized results are presented in Table S1. For the *INS* gene, the GPT 4.1 [38] model was able to correctly identify the provided DNA and amino acid sequences. Additionally, it accurately described all assigned molecular functions, biological process involvements, and the correct subcellular localization for *INS*. For longer sequences such as *BRCA1* and *IHH*, GPT 4.1 [38] claimed that it performed a BLAST [43] search which led it to the identification of the allegedly correct identification of the *BRCA1* sequence as *TP53* and *HTT*, while *IHH* was identified as *COL1A1* and *MPZ*. As shown in Table S1, the proteins identified by GPT 4.1 [38] for *BRCA1* and *IHH* do not exhibit significant sequence similarity to the reference sequences. This suggests that GPT 4.1 [38] may have difficulty interpreting sequence similarity in context, leading to the misidentification of these proteins. Next to issues with sequence similarities, the subsequent molecular functions were also partly incorrect, as *BRCA1* was describes as a protein involved in transport across membranes. The full responses are attached as an Excel sheet in additional file 2 called *“Case Study GPT-4.1”*.

### Genolator’s hidden states contain linguistically and biological plausible representations

**Fig. 3:**
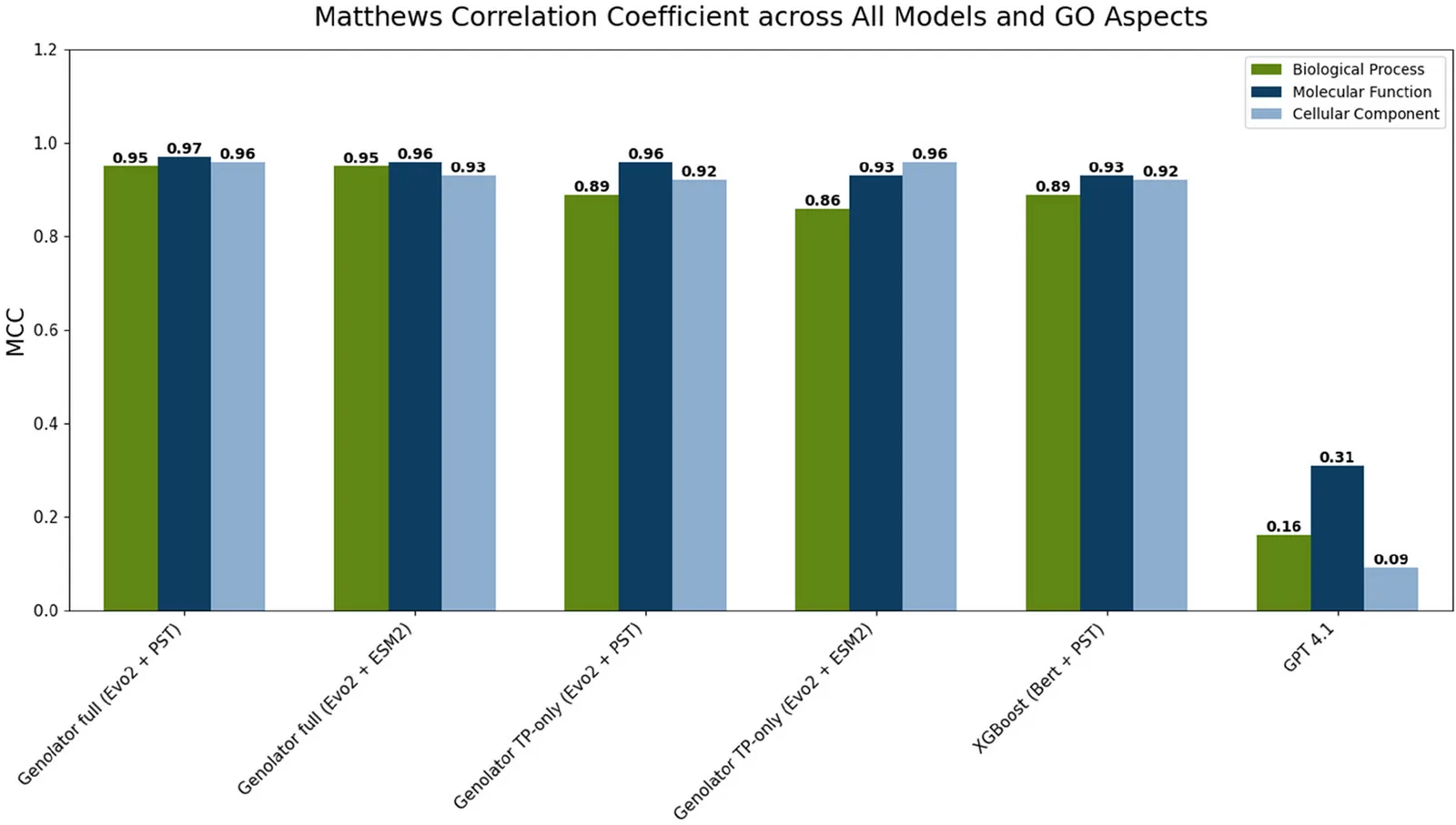
Bar plot displaying the Matthews Correlation Coefficient (MCC) achieved by each model across different GO aspects. The x-axis denotes the various models evaluated, while the y-axis indicates the MCC values. Each coloured bar represents a different GO aspect: Green – Biological Process, Dark Blue – Molecular Function, Light Blue – Cellular Component

**Fig. 4:**
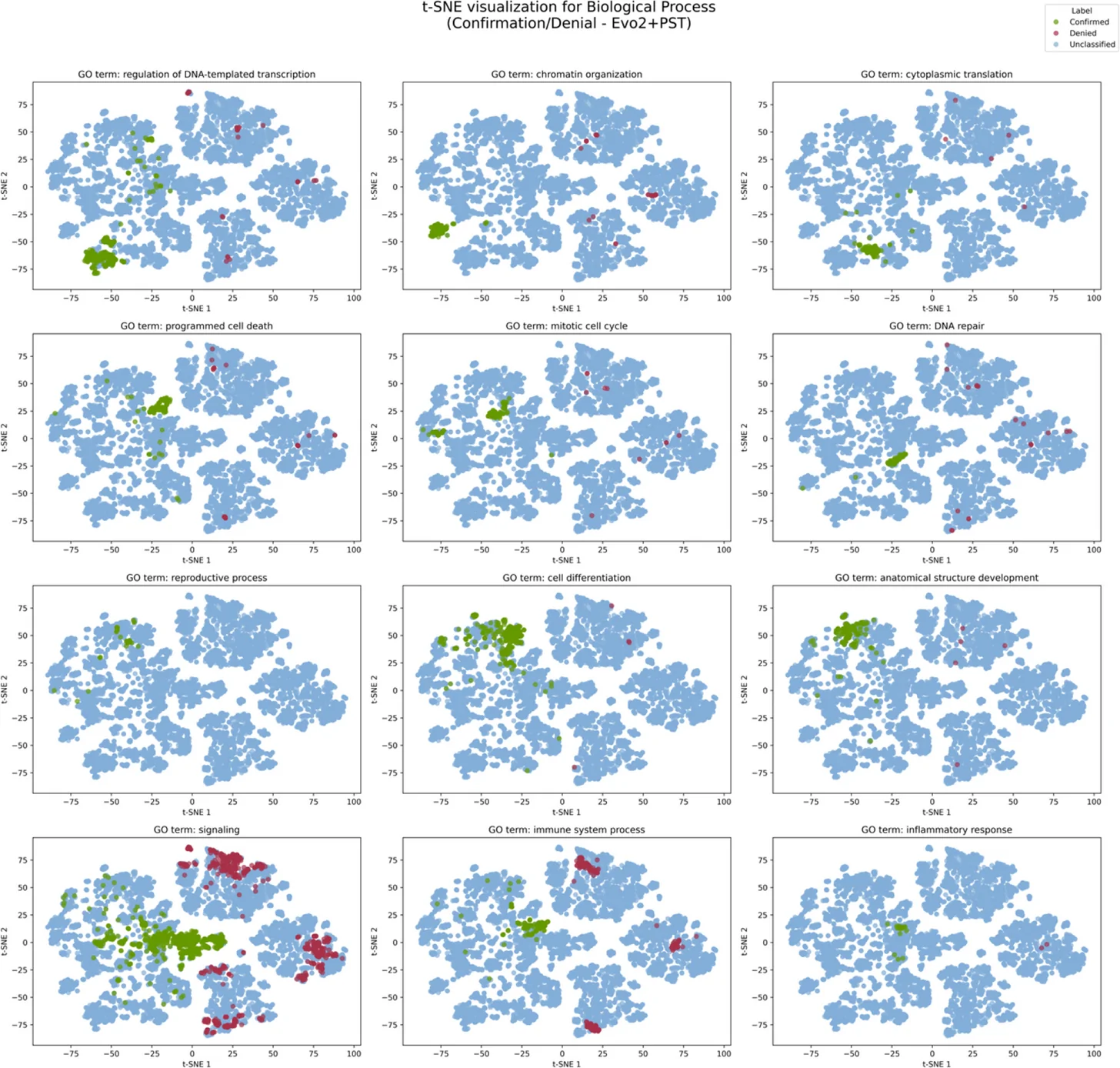
t-SNE visualization for a selection of biological process GO slim terms [29], generated using the last hidden states from the fine-tuned Llama [33] model. Each subplot represents a different GO term [29], displaying embeddings projected into two dimensions (t-SNE 1 and t-SNE 2 axes). Data points are coloured as follows: green (“Confirmed”) indicates the model associates the corresponding gene with the GO term [29]; burgundy (“Denied”) indicates the model denies the association; blue (“Unclassified”) indicates genes for which no association was queried

**Fig. 5:**
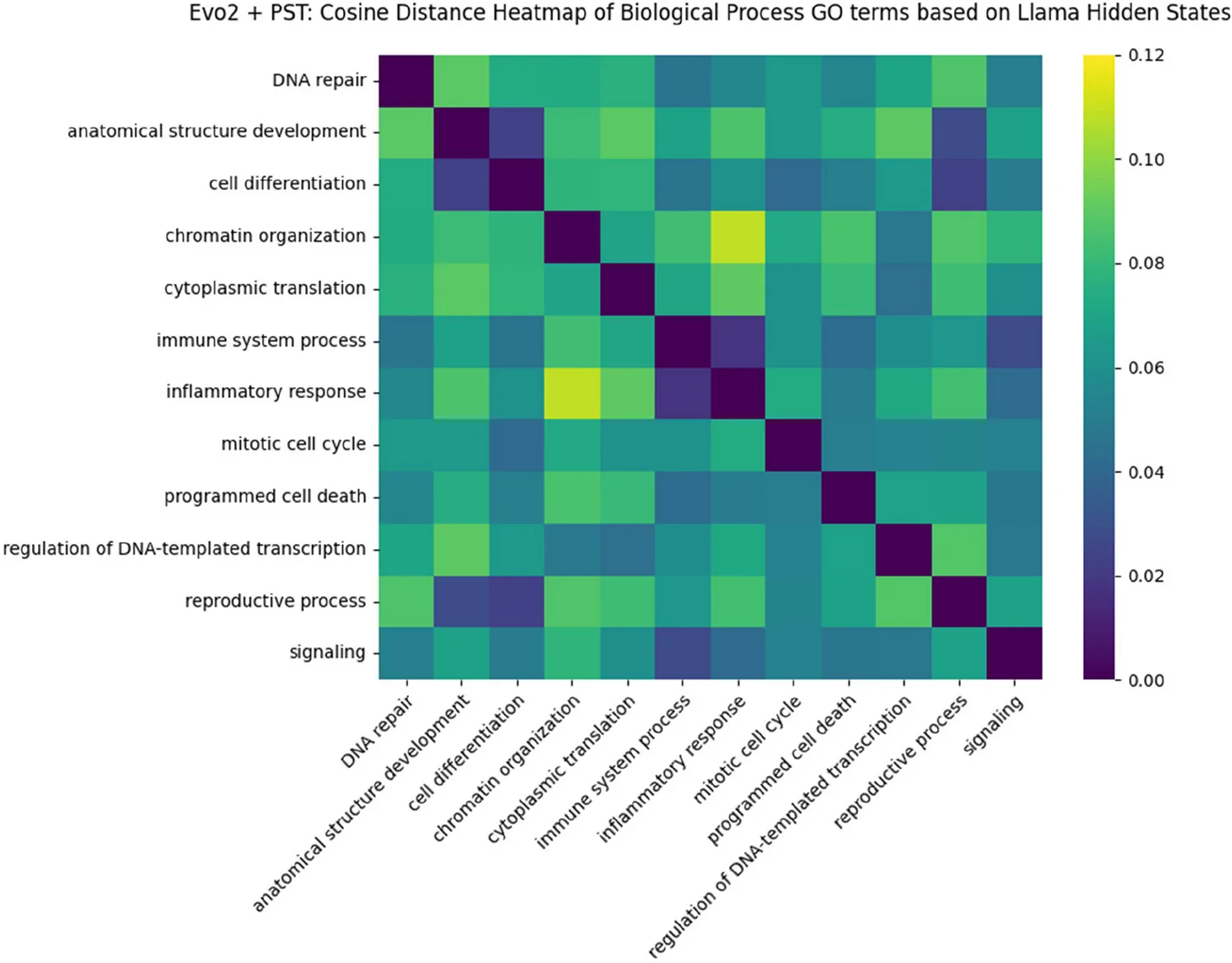
Cosine distance heatmap for a selection of biological process GO slim terms as represented in Llama-derived hidden state embeddings. Pairwise cosine distances were computed between embeddings of GO cellular component terms based on the hidden states from the Evo2 + PST model. Darker values represent greater similarity (lower cosine distance), while brighter values indicate greater dissimilarity. The heatmap visually highlights patterns of embedding similarity and potential functional relationships across biological processes

**Fig. 6:**
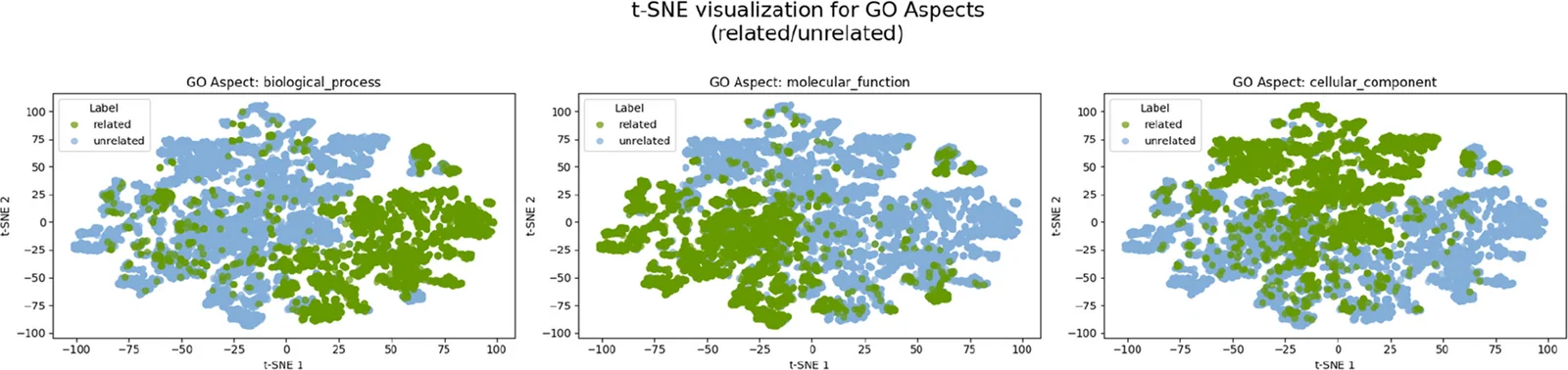
t-SNE [44] visualizations of the confirmation questions, generated from the last hidden states of the fine-tuned Llama model. Each subplot corresponds to one of the three GO aspects [29]. Green points represent datapoints related to relevant (confirmed) questions for the respective GO aspect [29], while blue points denote unrelated datapoints

To assess whether the model effectively integrated natural language and biological information, we investigated the features learned by Genolator. We extracted the hidden states from the final layer of the Llama [33] model prior to the output layer and projected them into a two-dimensional space using t-distributed stochastic neighbour embedding (t-SNE) [44]. This analysis was performed separately for each of the three GO-term [29] aspects, allowing us to visualize the distribution of hidden states according to whether the model confirmed or denied the queried association. Excerpts from the resulting visualizations can be seen in the Figs. 3, 4, 5 and 6. For most GO terms, Genolator achieved a clear separation between confirmed and denied associations in the t-SNE [44] space, producing distinct clusters across all GO-term aspects [29]. Additionally, within each GO aspect [29], Genolator clustered related terms in close proximity, while separating less related terms in the dimensionality reduced t-SNE space. For example, as visible in Figs. 3 and 4, for the association with biological processes the term “regulation of DNA-templated transcription” is directly neighboured by the term “chromatin organization”, a necessary preceding step for DNA translation, and the term “cytoplasmic translation”, a procedure following transcription. Cell cycle and cell death related processes were also clustered closely, neighboured by the term “DNA repair”. Similarly, developmental related biological processes were grouped together. Additionally, signalling and immune related biological processes were clustered accordingly. For the presented subset of the category molecular function, a similar observation was made, as visible in Figure S17. In the selected subset visible in Figure S17, we observed clustering between DNA and RNA related functionalities (“DNA binding”, “RNA binding”, “histone binding” and “transcription regulator activity”), structure related functionalities (“protein folding chaperones” and “structural molecule activity”), signalling and transport related functionalities (“transporter activity”, “receptor ligand activity” and “transducer activity”) and enzymes (“ligase activity”, “hydrolase activity” and “transferase activity”). Finally, for cellular components we again observed a plausible structuring of the hidden states, as visible in Figure S18. In this subset, nuclear compartment related localisations (“chromosome”, “nuclear chromosome”, “nucleolus”, “nucleus” and “nucleoplasm”) were grouped together, similarly to extracellular compartments (“extracellular matrix”, “extracellular region”, “extracellular space”).

**Fig. 7:**
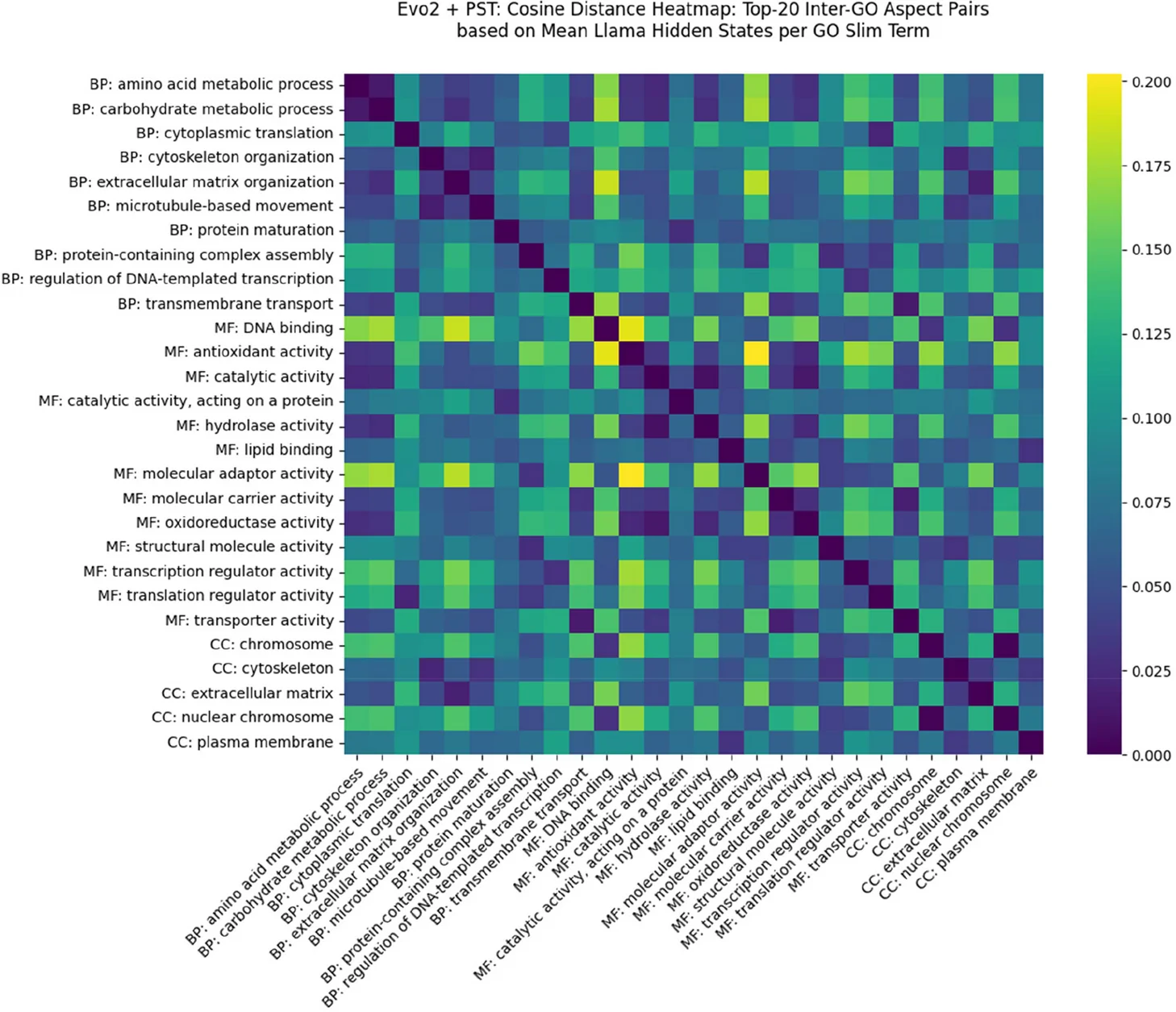
Cosine distance heatmap of the top-20 inter-GO aspect term pairs based on mean Llama hidden states from the Evo2 + PST model per GO slim term. Each row and column represents one of the 28 GO slim terms involved in the 20 most similar cross-aspect pairs, grouped by GO aspect (BP, MF, CC). Color intensity reflects cosine distance, where darker values indicate greater similarity between term representations. Only inter-GO aspect pairs were considered in the ranking; intra-aspect distances are included in the heatmap for contextual reference

**Fig. 8:**
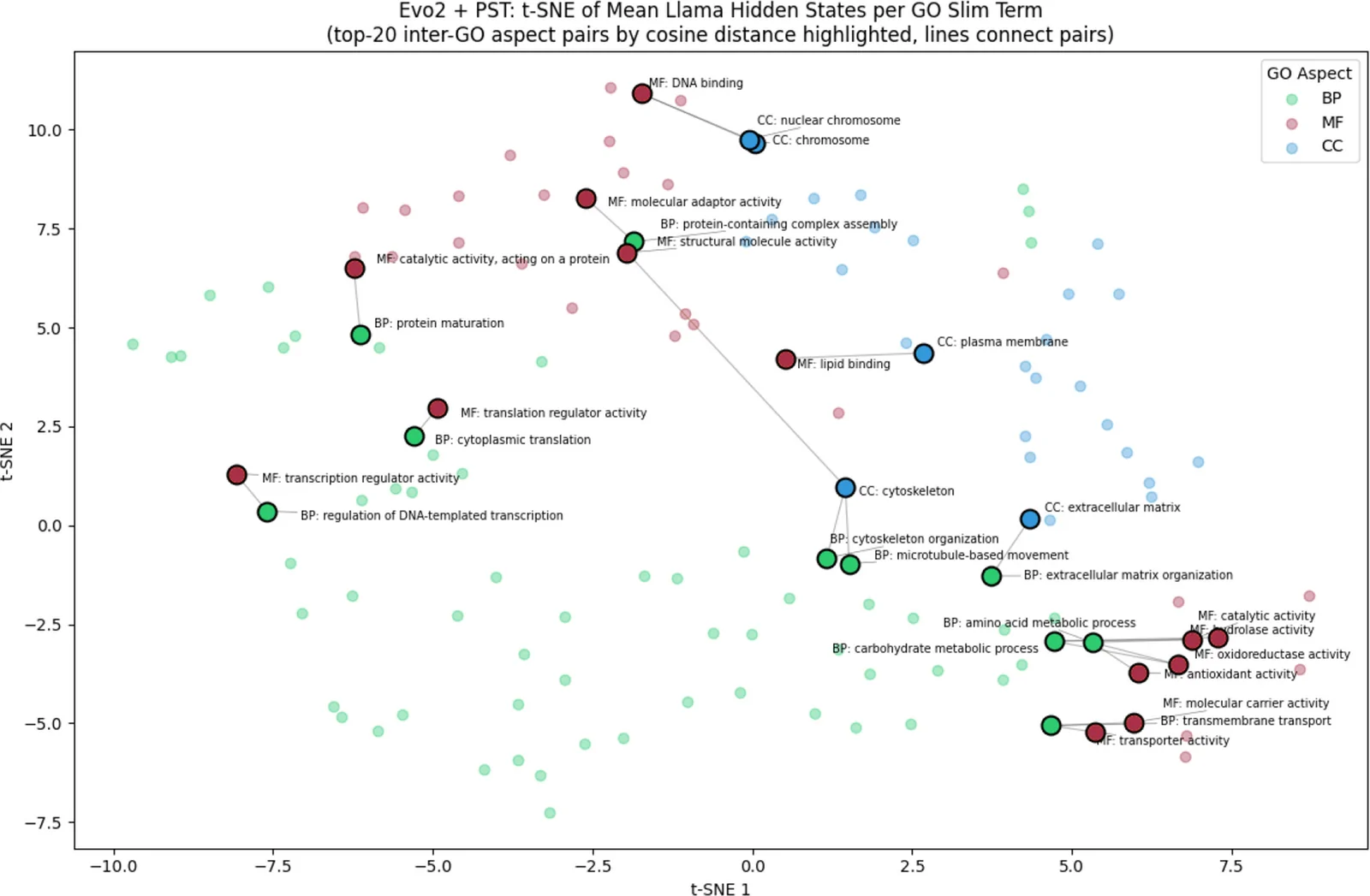
t-SNE projection of mean Llama hidden states from the Evo2 + PST model per GO slim term (133 terms total). Each point represents one GO slim term, coloured by GO aspect (green: biological process, burgundy: molecular function, blue: cellular component). Highlighted points (bold outline) indicate the GO slim terms involved in the top-20 most similar inter-GO aspect pairs by cosine distance; connecting lines link each pair. t-SNE was computed on the 133 × 4096 matrix of per-term averaged hidden states

**Fig. 9:**
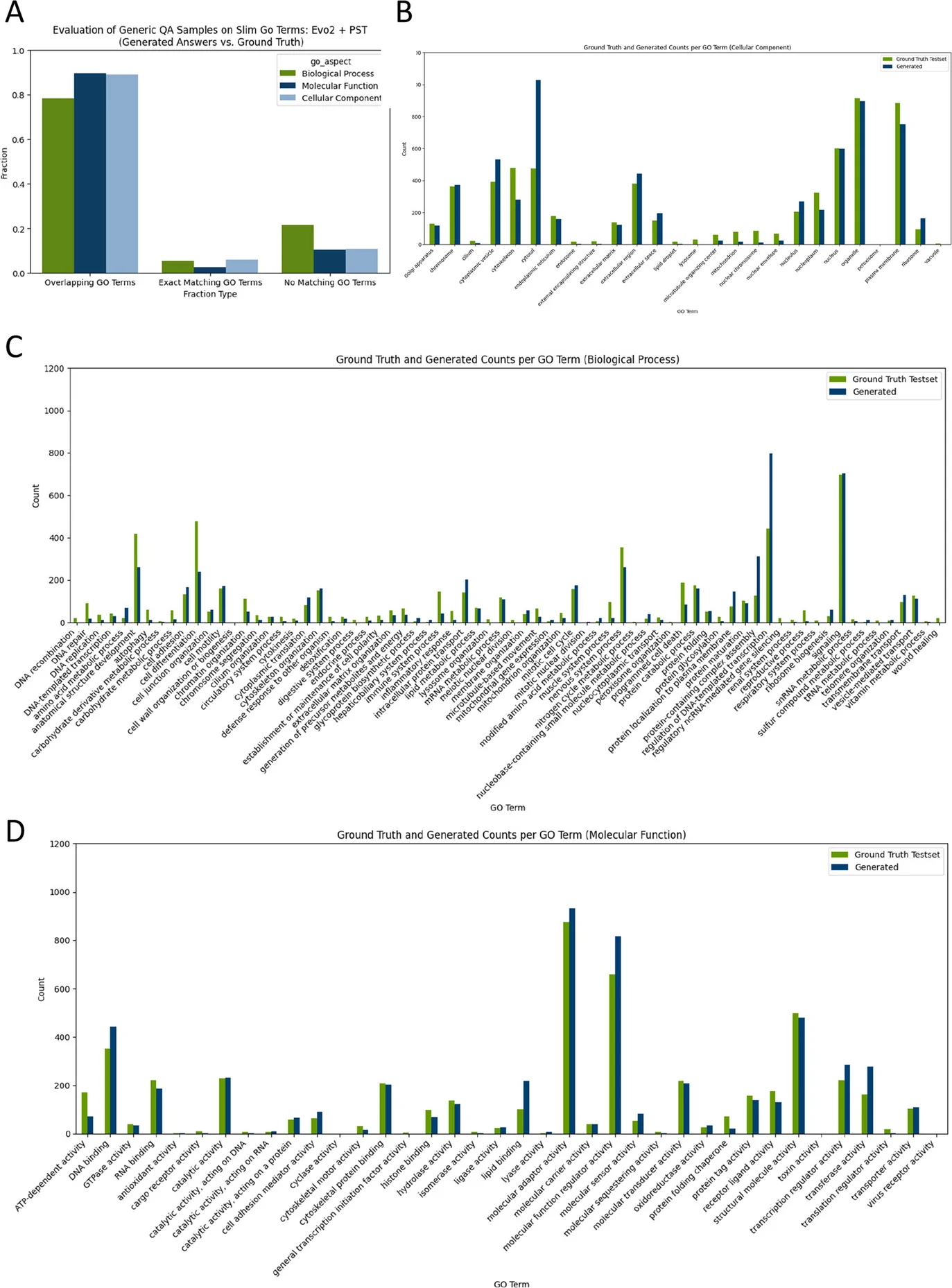
**A** Bar plot illustrating the evaluation of open question–answer (QA) samples on slim GO terms [29], comparing generated answers to ground truth annotations. The x-axis shows different fraction types: overlapping GO Terms [29], exact matching GO Terms [29], and no matching GO Term [29] for biological process aspect (green), molecular function aspect (dark blue) and cellular component aspect (light blue). **B** Bar plot showing the counts of ground truth and generated answers for each GO term [29] within the cellular component aspect. **C** Bar plot showing the counts of ground truth and generated answers for each GO term [29] within the biological process aspect. **D** Bar plot showing the counts of ground truth and generated answers for each GO term [29] within the molecular function aspect. For panels **B**–**D** each GO [29] term is displayed on the x-axis, with the corresponding sample counts on the y-axis (ranging from 0 to 1200). For each GO term [29], bars are grouped and color-coded: blue represents the ground truth counts and purple represents the Generated answer counts

To complement the patterns observed in the dimensionality-reduced t-SNE analysis, pairwise cosine distances were calculated among Llama-based hidden state embeddings across cellular component GO terms, preserving their full-dimensional relationships. The resulting heatmaps highlights relative similarities and dissimilarities between biological processes, molecular functions and cellular components supporting the spatial arrangements detected in the t-SNE projection and providing an additional quantitative assessment of the encoded semantic structure. These findings are visually represented in Figs. 7, 8 and 9.

The quantitative analysis of pairwise cosine similarities between biological process GO term embeddings yielded several clear patterns. The smallest cosine similarity values, indicating the strongest similarity within the embedding space, are observed between cell differentiation and anatomical structure development (cosine similarity = 0.023), as well as between immune system process and inflammatory response (cosine similarity = 0.019). Additionally, a low cosine similarity is evident between immune system process and signalling (cosine similarity = 0.027). In contrast, the cosine similarity between inflammatory response and chromatin organization is higher (cosine similarity = 0.108), representing a value approximately five times greater than those observed for the most similar term pairs. Although this value remains low in absolute terms, it is higher than most other pairwise similarities in the dataset. Pairwise similarities for a subset of biological process GO terms are visualized in Fig. 5.

The pairwise cosine similarities between molecular function GO terms notably strong similarity is observed between DNA binding and RNA binding (cosine similarity = 0.019), as well as between DNA binding and histone binding (cosine similarity = 0.028). A low cosine similarity is also present between histone binding and transcription regulator activity (cosine similarity = 0.045). Additionally, strong similarities are identified among the enzyme activity terms, including hydrolase activity and ligase activity (cosine similarity = 0.039) and between hydrolase activity and transferase activity (cosine similarity = 0.022). Transferase activity and ligase activity also display a low cosine similarity (cosine similarity = 0.023). Furthermore, a low cosine similarity is observed between transporter activity and molecular transducer activity (cosine similarity = 0.038).

In contrast, higher cosine similarities are quantified between transcription regulator activity and hydrolase activity (cosine similarity = 0.117), as well as between transporter activity and transcription regulator activity (cosine similarity = 0.112). In both cases, these values are several times higher than those observed between the most similar term pairs. A subset of pairwise cosine similarities for molecular function GO terms are visualized in Figure S19.

Higher cosine similarities for the pairwise analysis of cellular component GO terms, are observed particularly between chromosome and nucleus (cosine similarity = 0.021), cytoplasmic vesicle and extracellular region (cosine similarity = 0.043), cytoplasmic vesicle and cytosol (cosine similarity = 0.063), and cytosol and nucleoplasm (cosine similarity = 0.022).

In contrast, higher cosine similarity values are noted between chromosome and extracellular region (cosine similarity = 0.118), chromosome and extracellular space (cosine similarity = 0.117), as well as between nuclear chromosome and extracellular region (cosine similarity = 0.123) and nuclear chromosome and extracellular space (cosine similarity = 0.121), compared to the majority of the other term pairs. Pairwise values for a subset of cellular component terms are displayed in Figure S20.

### The Genolator implicitly learns the different categories of the GO-hierarchy

To further investigate Genolator’s general language and genomic understanding, we analysed its hidden states on sample level to assess whether the model could distinguish GO terms [29] belonging to different aspects. As shown in Fig. 6, Genolator tends to form three largely separable clusters, each corresponding to one of the three GO aspects [29]. However, overlaps between the three GO aspects were visible and investigated in more detail by computing the pairwise inter-GO aspect cosine distance based on the mean Llama hidden states per GO slim term, with results visualized as a heatmap in Fig. 7. The same aggregated hidden states were further used to generate a GO term-level t-SNE projection (Fig. 8), in which the same three-cluster separation, one per GO aspect, can be identified, consistent with the sample-level t-SNE plot in Fig. 6. The pairwise inter-GO aspect cosine similarities reveal that the lowest values, indicating the strongest cross-aspect similarity, are found predominantly between biological process (BP) terms and their directly associated molecular functions (MF) or cellular components (CC). The lowest inter-aspect cosine similarity is observed between transmembrane transport (BP) and transporter activity (MF) (cosine similarity = 0.015), reflecting the direct definitional relationship between the process and its underlying molecular function. Similarly low cosine similarities are found between extracellular matrix organization (BP) and extracellular matrix (CC) (cosine similarity = 0.018), cytoplasmic translation (BP) and translation regulator activity (MF) (cosine similarity = 0.021), and cytoskeleton organization (BP) and cytoskeleton (CC) (cosine similarity = 0.022). Among BP–MF pairs, amino acid metabolic process and carbohydrate metabolic process show consistently low cosine similarities to catalytic activity (MF) (cosine similarity = 0.023 and 0.025, respectively), as well as to hydrolase activity (MF) (cosine similarity = 0.027) and oxidoreductase activity (MF) (cosine similarity = 0.027 and 0.030). Further low cosine similarities are observed between protein-containing complex assembly (BP) and structural molecule activity (MF) (cosine similarity = 0.027), protein maturation (BP) and catalytic activity acting on a protein (MF) (cosine similarity = 0.027), and regulation of DNA-templated transcription (BP) and transcription regulator activity (MF) (cosine similarity = 0.029). Cross-aspect MF–CC associations are captured between DNA binding (MF) and nuclear chromosome (CC) (cosine similarity = 0.029) and chromosome (CC) (cosine similarity = 0.030), as well as between lipid binding (MF) and plasma membrane (CC) (cosine similarity = 0.030) and structural molecule activity (MF) and cytoskeleton (CC) (cosine similarity = 0.030). In contrast, within the selected subset, the highest inter-aspect cosine similarities are observed between DNA binding (MF) and extracellular matrix organization (BP) (cosine similarity = 0.185), molecular adaptor activity (MF) and extracellular matrix organization (BP) (cosine similarity = 0.181), molecular adaptor activity (MF) and carbohydrate metabolic process (BP) (cosine similarity = 0.176), and DNA binding (MF) and carbohydrate metabolic process (BP) (cosine similarity = 0.175), reflecting the biological distance between nuclear regulatory functions and extracellular or general metabolic processes.

### Asking the Genolator openly formulated questions about gene functionality

Next to understanding the confirmation or denial of associations between GO aspects [29] and specific proteins, we also trained Genolator to answer open-ended questions about GO aspects [29]. This open-ended, more generic capability is particularly valuable for proteins whose involvement in certain GO aspects is currently unclear or poorly characterized (“uncharacterized proteins”). For this we created a separate QA-dataset. We mapped all GO aspects to the slim GO terms using a GPT 4.1 [29] model, thereby enabling the evaluation of the overlap between the predicted and ground truth values. While the original hierarchy for the ground truth GO terms was available, we still decided to keep this mapping approach for the ground truth labels and the predicted labels, to be more consistent in the evaluation. Evaluation was performed using the best-performing model Genolator (Evo2 + PST/Full Fine-Tuning), as determined from binary classification results. For each sample, results were categorized into three groups:

The first group included samples with an exact match, where all ground truth functional terms were correctly predicted, and no additional terms were introduced. The second group comprised samples with partial overlap—some, but not all, ground truth terms were predicted, or additional, irrelevant terms were included. The final group contained samples in which only terms absent from the ground truth were predicted. The visualisation for the three GO aspects [29] can be seen in Fig. 9A. For generic questions regarding the involvement in biological processes 5.44% perfect matches were achieved, 78.51% matches showed an overlap with the ground truth and 21.49% showed a complete dissonance between the prediction and the ground truth. For the prediction of the cellular component 5.83% perfect matches were achieved, 89.19% showed an overlap with the ground truth and 10.81% were in complete disagreement with the ground truth. Finally, for the prediction of the molecular function, 2.51% of the samples showed a perfect match, 89.59% showed an overlap with the ground truth and 10.41% were in complete disagreement.

In the cellular component aspect (Fig. 9B), the overall distribution of GO term slim counts in Genolator’s generated predictions closely matches that of the ground truth data. Several terms show strong concordance, including chromosome (ground truth: 363; generated: 372), Golgi apparatus (ground truth: 129; generated: 117), nucleus (ground truth: 601; generated: 598), and organelle (ground truth: 915; generated: 897). This alignment reflects model effectiveness in reproducing the relative frequencies of these key cellular component terms.

An overshoot in counts is observed for cytosol (ground truth: 473; generated: 1030) and cytoplasmic vesicle (ground truth: 393; generated: 532). Conversely, plasma membrane (ground truth: 885; generated: 752) is under-represented in the generated outputs. Less frequent terms such as lipid droplet, lysosome, peroxisome, and vacuole remain rare in both datasets.

Overall, Genolator’s generated answers recapitulate the qualitative distribution of cellular component GO terms, with strong agreement for many major components and some divergence in individual term frequencies.

In the biological process aspect (Fig. 9C), both the ground truth and Genolator’s generated predictions most prominently feature the term signalling (ground truth: 696; generated: 704), with counts closely matched. Similarly, several other terms show good agreement, including mitotic cell cycle (ground truth: 157; generated: 175), cell motility (ground truth: 161; generated: 171), cell adhesion (ground truth: 132; generated: 167), and protein catabolic process (ground truth: 175; generated: 160). Rare terms, such as DNA recombination, meiotic nuclear division, and tRNA metabolic process, remain uncommon in both datasets.

Nonetheless, the overall distribution reveals several notable disparities. Terms such as anatomical structure development (ground truth: 418; generated: 260), cell differentiation (ground truth: 477; generated: 238), and regulation of DNA-templated transcription (ground truth: 444; generated: 97) are predicted considerably less often than their ground truth counts. Additionally, a marked under-representation is seen for programmed cell death (ground truth: 189; generated: 84) and immune system process (ground truth: 147; generated: 44).

Other terms, including protein-containing complex assembly (ground truth: 312; generated: 128), protein localization to plasma membrane (ground truth: 219; generated: 147), and extracellular matrix organization (ground truth: 58; generated: 33), exhibit lower counts in the generated outputs.

Overall, while Genolator’s predictions recapitulate the qualitative frequency pattern for a subset of biological process GO terms particularly for more frequent and rare terms the correspondence between predicted and ground truth distributions for this aspect remains less well matched than for cellular component terms.

In the Molecular Function aspect (Fig. 9D), the overall distribution of GO term counts in Genolator’s predictions closely matches the ground truth data. The most frequent terms in both datasets are molecular adaptor activity (ground truth: 877; generated: 932) and molecular function regulator activity (ground truth: 659; generated: 816). Several additional terms show particularly close agreement, including catalytic activity (ground truth: 229; generated: 232), molecular carrier activity (ground truth: 38; generated: 38), structural molecule activity (ground truth: 499; generated: 479), transporter activity (ground truth: 102; generated: 111), ligase activity (ground truth: 25; generated: 27), GTPase activity (ground truth: 39; generated: 33), and protein tag activity (ground truth: 157; generated: 141).

Some terms exhibit notable discrepancies, including lipid binding (ground truth: 101; generated: 218) and transferase activity (ground truth: 162; generated: 278), which are substantially over-represented in the generated set. Differences are also observed for transcription regulator activity (ground truth: 221; generated: 284) and protein folding chaperone (ground truth: 72; generated: 22).

Rare terms, such as antioxidant activity (ground truth: 1; generated: 1), remain infrequent in both datasets.

Overall, Genolator’s output recapitulates the qualitative distribution of molecular function GO terms, with strong agreement for many major categories and isolated cases of under- or over-representation among specific terms. The test set for the open questions is attached as a Excel sheet in Additional file 2 called *“KMeans Split Test Set”.*

### Analysis of attention patterns across open-ended questions

**Fig. 10:**
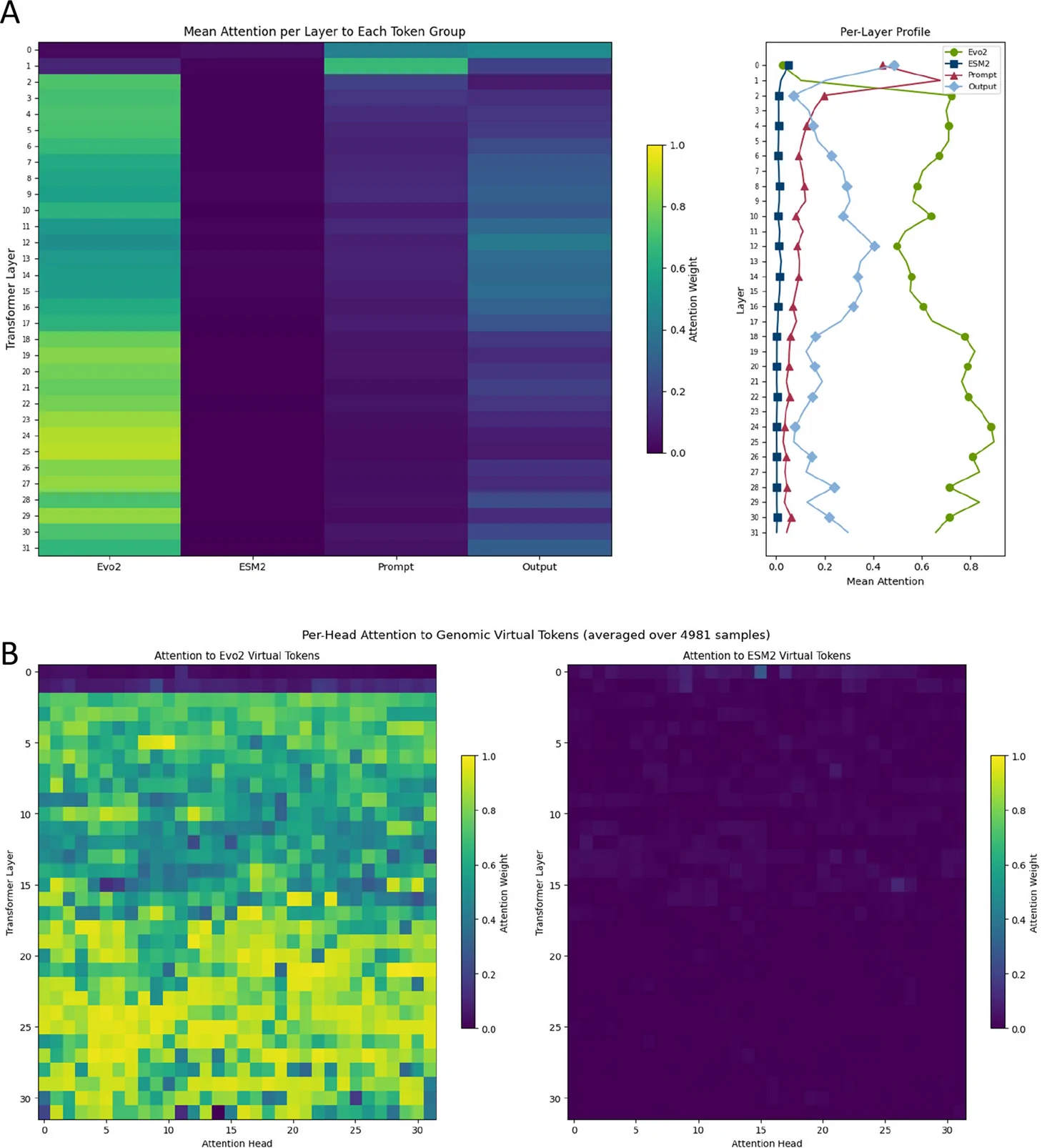
Layer- and Head-Wise Attention Genolator (Evo2 + ESM2/Full Fine-Tuning). **A** Mean attention weights to each token group (Evo2, ESM2, prompt, output) across all transformer layers, visualized both as a per-layer heatmap and as per-layer profile plots. This panel illustrates how the model’s attention is distributed among DNA (Evo2) and protein sequence (ESM2) tokens, as well as prompt/output tokens, throughout model depth. **B** Per-layer, per-head attention heatmaps for Evo2 and ESM2 tokens, revealing patterns of specialization among attention heads. Elevated attention in certain heads indicates that individual attention heads may focus strongly on specific modalities, even when the averaged layer attention is low. Colour scale: Attention values range from 0 (dark blue/purple, low attention) to 1 (yellow, high attention). All results are averaged across the test dataset

**Fig. 11:**
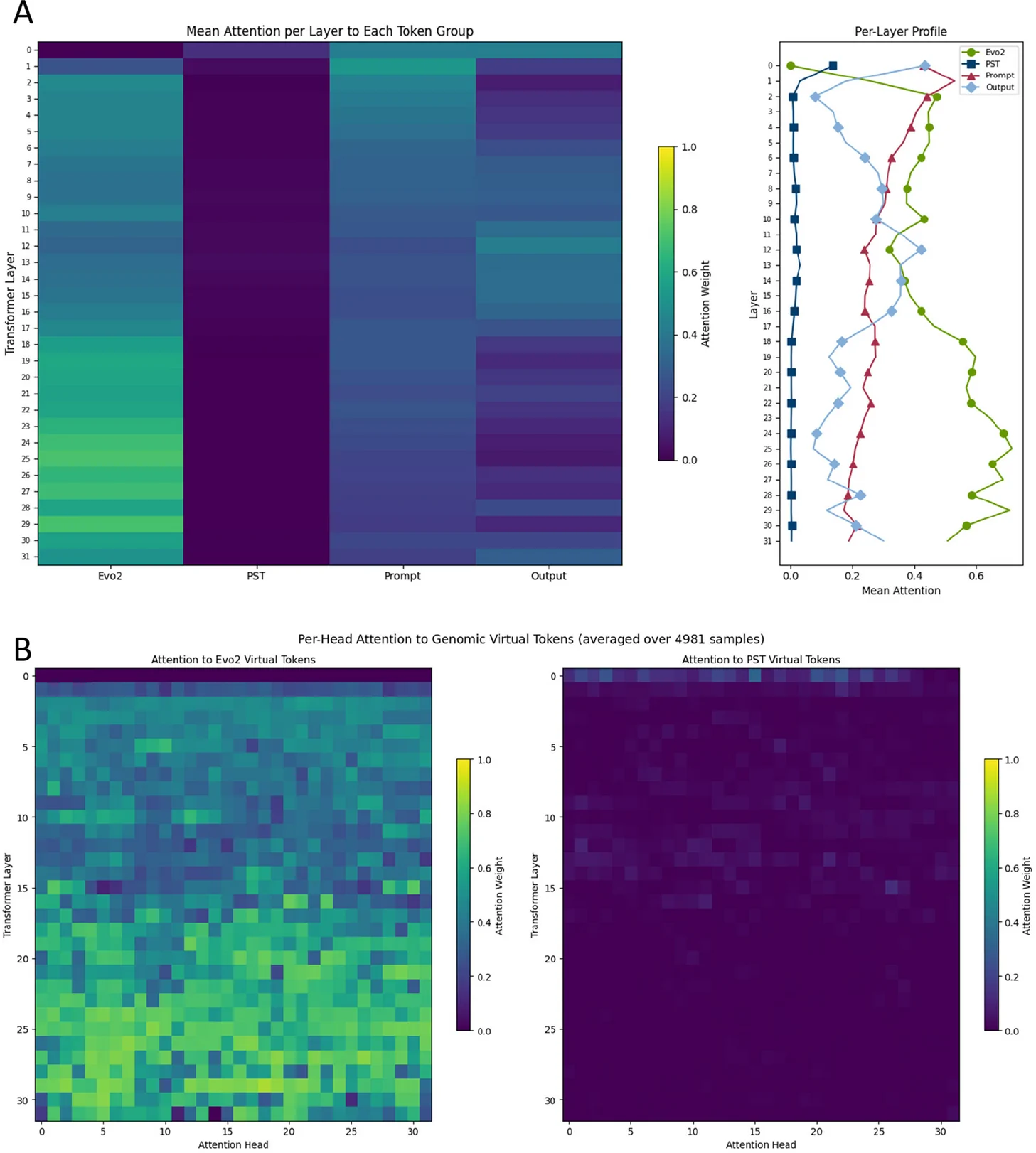
Layer- and Head-Wise Attention Genolator (Evo2 + PST/Full Fine-Tuning). **A** Mean attention weights to each token group (Evo2, PST, prompt, output) across all transformer layers, visualized both as a per-layer heatmap and as per-layer profile plots. This panel illustrates how the model’s attention is distributed among DNA (Evo2) and structural enriched protein sequence (PST) tokens, as well as prompt/output tokens, throughout model depth. **B** Per-layer, per-head attention heatmaps for Evo2 and PST tokens, revealing patterns of specialization among attention heads. Elevated attention in certain heads indicates that individual attention heads may focus strongly on specific modalities, even when the averaged layer attention is low. Colour scale: attention values range from 0 (dark blue/purple, low attention) to 1 (yellow, high attention). All results are averaged across the test dataset

To assess the potential benefits of combining Evo2, ESM2, and PST embeddings, we analysed how multimodal integration influenced Genolator’s predictions for open-ended questions by evaluating the model’s attention weights across modalities and GO aspects. Specifically, attention weights across all 32 Llama layers were examined. For both models, the mean attention for each group of tokens (Evo2, ESM2/PST, prompt, output) was calculated per layer (Figs. 10A, 11A). Additionally, per-head attention weights to each group of tokens were computed for detailed analysis, focusing on genomic virtual tokens (Figs. 10B, 11B).

#### Genolator (Evo2 + ESM2/full fine-tuning)

Mean attention analysis revealed that attention to ESM2 virtual tokens was generally low across all layers reaching a maximum mean per-layer value of 0.050 (layer 0) while Evo2 virtual tokens received the highest attention after the initial layers. Specifically, from layers 2 to 31, Evo2 mean attention exceeded 0.6 for most layers, peaking in layer 29 with a value of 0.837. The highest per-head attention to Evo2 tokens was 0.99 (layer 21, head 29). Prompt and output token attention was highest in layers 0 and 1 (up to 0.68 and 0.49, respectively) and decreased in deeper layers. Attention to ESM2 tokens was generally low in all but the first layer, with the highest per-head ESM2 attention at 0.28 (layer 0, head 15). The mean per-layer maximum for Evo2 virtual tokens was 0.765.

#### Genolator (Evo2 + PST/full fine-tuning)

In contrast, the Evo2 + PST model exhibited distinct patterns. In the first layer, mean attention to PST virtual tokens was 0.138, with the maximum per-head PST attention reaching 0.32 (layer 0, head 15). The attention to PST tokens in layer 1 was not uniformly distributed across all attention heads. Rather, multiple heads showed elevated attention, with several heads exceeding 0.20 (e.g., heads 1, 3, 12, 15, 20, 22), and the majority of heads having values greater than 0.10. In the same layer, prompt and output tokens had mean attention values of 0.43 each, and Evo2 tokens showed negligible attention (0.0004). Evo2 attention increased over subsequent layers, peaking at 0.708 in layer 29 (per-layer mean), with the highest per-head attention in this layer as well (0.87, head 18). For PST tokens, attention declined sharply after the first layer (mean < 0.01 from layer 2 onward). Prompt and output tokens maintained relatively stable attention from layers 1 to 31 (mean 0.20–0.30). These results quantitatively demonstrate a shifting attention focus across the Llama architecture in multimodal models.

### Virtual token ablation study on trained models

To further estimate the importance of the virtual tokens representing each modality, we performed a token ablation study for both the Evo2 + ESM2 and Evo2 + PST models. For this analysis, virtual tokens were replaced with zero vectors, either one modality at a time or both simultaneously, and the cross-entropy loss was computed on the open-ended test set.

**Fig. 12:**
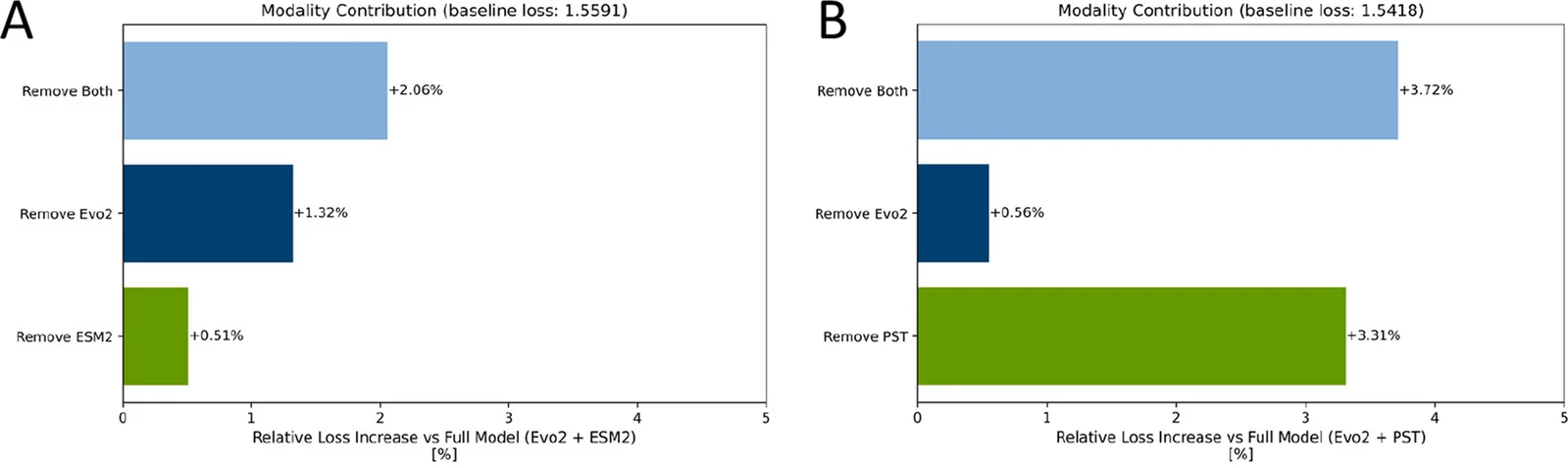
**A** Evo2 + ESM2 Model: bars depict the relative increase in test set loss upon removal of individual or both virtual token modalities compared to the full model baseline. Green indicates loss increase from ablating ESM2 tokens, dark blue from ablating Evo2 tokens, and light blue from ablating both modalities together. **B** Evo2 + PST model: equivalent results for the Evo2 + PST model, with green for PST, dark blue for Evo2, and light blue for both. A greater loss increase indicates higher modality importance for accurate prediction. For comparison: the difference between first and last training validation loss was around 3.6%

For the Evo2 + ESM2 model (Fig. 12A), the baseline test loss was 1.5591. Removing the Evo2 tokens led to a relative increase in loss of 1.32%, while removing ESM2 tokens resulted in a smaller increase of 0.51%. Ablating both Evo2 and ESM2 tokens together led to a total loss increase of 2.06% compared to the baseline.

For the Evo2 + PST model (Fig. 12B), the baseline test loss was slightly lower at 1.5418. In this model, removing Evo2 tokens resulted in a 0.56% loss increase, while ablating PST tokens increased the loss by 3.31%. Simultaneous ablation of both modalities led to a 3.72% increase in loss. 

These results indicate that, in the Evo2 + PST model, PST tokens contribute substantially more to model performance than Evo2 tokens, while in the Evo2 + ESM2 model, Evo2 tokens have a greater impact than ESM2 tokens.

## Discussion

### The Genolator – a multimodal language model for protein function interpretation

Here we present the Genolator, a finetuned Llama [33] model capable of giving robust evaluations whether a specific protein is associated with functionality terms derived from the three GO aspects [29] cellular localisation, molecular function and involvement in biological processes. We demonstrate that integrating a large language model capable of processing natural language with genomic embeddings from multimodal models yields a system that outperforms both general-purpose LLMs such as GPT 4.1 [38] and smaller, domain-specific models such as an XGBoost [41] classifier trained on the PST embeddings. The resulting Genolator can interpret genomic embeddings based on instructions given in English, thereby making the use of embeddings from large genomic models more accessible and paving the way for a new, language-driven interface for interacting with genomic data. Furthermore, by fusing natural language with genomic code, Genolator is able to learn interpretable representations that cluster related GO terms [29] in a biologically meaningful manner. This plausibility is maintained not only for linguistically similar terms but also for biologically related processes and compartments, highlighting the potential of natural language as a powerful tool for exploring gene function. Previously it has been demonstrated that the prediction of GO-terms [29] can be improved by including explainable text descriptions to the predicting model [7, 9]. The development and evaluation of Genolator builds upon this evidence, as its strong performance on confirmative and denial questions, as well as the analysis of its hidden states in these contexts, provide additional support for the value of integrating language-based explanations in functional genomics prediction. Consistent with previous reports, we found that predicting the association between a genomic element and a biological process was the most challenging aspect of gene functionality prediction [7]. Further studies are required to transfer the Genolators applicability to multiple species and observe how this ability is influenced by proteins from multiple species being present in the training data.

### The Genolator outperforms generalist and specialist baseline models

We provide extensive evidence that openly available LLMs such as GPT 4.1 [38] are currently inadequate for processing genomic sequences and truly understanding the underlying genomic context; their capabilities are limited to natural language-based comprehension. On top of this, these models often generate responses with extensive explanations and may claim to use genomic analysis tools such as BLAST [43] to lend credibility to their answers, even when their outputs are incorrect or contain hallucinated elements, including fabricated BLAST [43] results. Additionally, with its large context length (1.000.000 tokens), it should have the technical capability to process large genomic sequences. However, in our small case study we observed that GPT 4.1 [38] only handles small proteins well (such *INS*), for which it might remembered the complete amino acid sequence. For longer proteins such as *BRCA1*, it does not only retrieve dissimilar proteins (as indicated by the non-significant overlap returned by BLAST [43]) but also suggest candidates which are dissimilar in the simplest aspects such as the amino acid length (*BRCA1* has around 1800 amino acids, *HTT* has around 3000 amino acids). Combining all this evidence with its poor performance on the binary classification task (MCC = 0.18) suggests that GPT 4.1 [38] struggles with the analysis of genomic sequences. These findings are particularly intriguing given that GPT 4.1 [38] has likely encountered a substantial number of genomic sequences during its training. This observation underscores that—as already stated—despite such exposure, its capabilities remain limited to natural language-based comprehension rather than a true understanding of genomic context. This highlights the need for multimodal language models with a more nuanced grasp of genomic information such as Genolator.

In addition to GPT 4.1, we compared Genolator to an XGBoost [41] model, which was provided with the PST [37] embeddings as well as prompt embeddings from a BioBERT [42] sentence transformer. Although Genolator outperformed the XGBoost [41] model across all GO-term categories, the advantage of Genolator extends well beyond this modest performance margin. By leveraging a generative model like Llama, Genolator enables natural language querying, making the tool more accessible and facilitating the use of natural language to explore its learned representations. Moreover, as a generative architecture, Genolator can serve as a true multitask model and is not limited to binary classification tasks commonly addressed with models such as XGBoost. This flexibility is illustrated by Genolator’s design, which incorporates the capacity to address open-ended questions and generate complex, context-aware responses enabling applications beyond those achievable with traditional discriminative classifiers. While performance on such tasks is not yet optimal, the model exhibits more than promising results, as discussed in a later section. It should also be noted that, for cost reasons, the Llama-based Genolator was not subjected to hyperparameter tuning, while the XGBoost [41] model did undergo tuning, giving the latter a slight advantage in terms of optimization. Tuning Genolator’s hyperparameters and playing with the number of virtual tokens offers significant potential for further performance improvement. Additionally, architectural design choices such as the configuration of the token projectors and the strategy used for token fusion are also anticipated to contribute to further enhancements. Moreover, replacing the Llama [33] backbone with larger, state-of-the-art language models (e.g. GPT 5 [45, 46] or Gemini [47, 48]) could also provide additional benefits.

### Differentiating to existing models

The field of integrating genomic language models with natural language models is still emerging. Contrary to existing protein prediction models, Genolator is a generative model, instead of a pure classification or regression model, which increases user accessibility and allows shared analysis of natural and genomic language. To our knowledge, ChatNT [47]—a generalist conversational agent that leverages the nucleotide transformer model to address various binary and regression tasks involving genomic features and elements—is the only currently available model that builds on the related concept of multimodal generative models in the field of genomics. However, ChatNT [28] has not been trained to predict gene functionality with respect to GO aspects [29] and does not provide the capability to incorporate amino acid embeddings or protein structure information. Furthermore, Evo2 [22], the model used to generate DNA embeddings for Genolator, offers a substantially larger input size (1 million) compared to the nucleotide transformer model (12 kb) [24]. This was especially advantageous for Genolator, as it enabled the encoding of complete cDNA regions, including UTR regions. Furthermore, evidence from the literature provides hints that models with larger input size can outperform models with smaller input sizes, in coding and non-coding tasks [22, 49]. Both increasing the sequence context window and integrating embeddings for amino acid sequences and protein structure have been identified as important future directions by the creators of ChatNT [28]; however, these features are currently absent from their model. The two approaches also differ fundamentally in their training strategies. In Genolator, only the token projectors and the Llama [33] model were trained/fine-tuned, while all embedding models—including Evo2 [22], ESM2 [36], and the PST [30]—remained frozen throughout training. By contrast, ChatNT [28] trains both its projection model and its nucleotide encoder (analogous to Genolator’s use of Evo2 [22] for the DNA embedding step), but keeps the English decoder frozen.

A key advantage of our approach is the demonstrated value of foundation models like Evo2 [22], ESM2 and PST, which can generate highly informative embeddings for downstream tasks without requiring additional fine-tuning. This flexibility allows for rapid migration or replacement of embedding models, as well as straightforward retraining. The strong performance of our XGBoost [41] baseline, trained directly on these structurally enriched protein sequence embeddings, further underscores their quality.

Additionally, we evaluated an ablated version of Genolator in which only the token projectors were trained while keeping the Llama [33] backbone frozen. Although this projector-only model underperformed relative to the fully fine-tuned Genolator and performed similarly compared to the XGBoost [41] model, it still achieved remarkably good results suggesting a knowledge distillation-like [50] effect. This is particularly noteworthy, as it enables efficient migration and experimentation with larger LLMs for which only the token projectors need to be retrained.

One limitation of our approach is that neither the architecture nor the hyperparameters of the token projectors (nor those of the Llama model in the case of the full Genolator) were subject to extensive tuning, which suggests further room for optimization in future work. Further studies will be necessary to systematically compare these different strategies and to establish optimal training protocols for multimodal natural language and genomic language models. 

Finally, it is important to note that ChatNT [28] is specifically designed for targeted binary classification tasks (such as detecting the presence of genomic elements like lncRNAs) and regression tasks (such as predicting protein stability), where it demonstrates strong performance. However, it is not intended to answer generic, open-ended natural language questions. This specialized focus allows ChatNT [28] to Excel in well-defined prediction scenarios, while also underscoring the need for further research to explore the capabilities and adaptability of generalist genomic conversational agents in addressing open-ended functional genomics queries for which we demonstrate a first impulse using Genolator.

### Evaluating the Genolators capability to answer open-ended questions

Training a large language model to answer generic open-ended questions, rather than confirming or denying specific functional associations, broadens the range of possible applications. However, it comes with some hurdles. One challenge is to assess the quality of the generated responses. In general, it is possible to evaluate the predictions made by employing experts from the relevant field, this however was not realistically possible for a project like this in which multiple thousands of predictions are made encompassing a wide topic, due to limited personnel. To address these challenges, we evaluated Genolator’s (Evo2 + PST/Full training) predictions from multiple perspectives to provide a comprehensive overview of its capabilities in answering generic open-ended questions. LLMs are commonly evaluated using cross entropy and perplexity [51]. While perplexity is a standard metric for evaluating language models, it has significant limitations in open-ended tasks such as answering generic questions about biological processes, molecular functions, and cellular components of a given sequence. Perplexity and cross-entropy do not directly capture the quality or correctness of generated responses. This challenge is reflected in recent findings that perplexity, while widely used as a proxy for evaluation, cannot be directly linked to factuality in generation settings, as it is affected by many linguistic phenomena and by the variability of valid responses [52–54]. The issue is particularly pronounced for proteins with a wide range of known functions, as the ground truth dataset contains many associated labels, making it considerably more challenging for the model to identify every single label. Moreover, the specific phrasing of a response can impact both perplexity and cross-entropy, since different textual descriptions of the same function may not be evaluated fairly by these metrics—a limitation observed in several cases produced by Genolator.

To address these shortcomings, we implemented a GPT 4.1-based LLM-as-judge approach to map both Genolator’s predictions and the ground truth annotations to a reduced set of GO Slim terms [29], thereby reducing evaluation complexity and enhancing comparability. Additionally, when constructing the training samples, we simplified the ground truth GO term descriptions to focus only on the primary GO terms for each gene, facilitating fairer and more consistent assessments. However, this approach may sometimes disadvantage Genolator during evaluation, as it is possible for the model to correctly predict legitimate GO terms for a gene that were not included in the shortened ground truth annotations. Overall, while perplexity remains useful for intra-model comparisons, a more nuanced and factuality-aware evaluation is essential for reliable assessment and continued improvement of model and dataset quality in open-ended biological tasks.

We observed that, for most questions regarding proteins in the test dataset, Genolator was able to predict functionalities that overlapped with the ground truth to a high degree (biological processes: 78.51%; molecular function: 89.59%; cellular component: 89.19%). The comparison of predicted and ground truth term counts demonstrates that Genolator effectively captures the relative distribution of GO Slim terms [29] for cellular component and molecular function, closely reflecting the empirical label frequencies. While recapitulating comparable frequencies of GO terms does not necessarily reflect correctness of predictions, it demonstrates the Genolators capabilities and systemic coverage across the analysed GO spectrum. Especially, in the biological process aspect, some divergence is still observed, reflecting the greater complexity and diversity of these functional categories. These results highlight Genolator’s strong capability to answer open-ended questions and its potential as a valuable tool for functional genomics research. Remaining challenges emphasize the need for further methodological development and robust evaluation strategies for open-ended biological prediction tasks. Importantly, our analysis brings to light a critical challenge in modelling and evaluating protein function: the significant underrepresentation of certain GO terms [29] in the available data. This data imbalance not only complicates fair evaluation but may also limit the model’s ability to generalize to rare or less frequently annotated functions. An additional benefit of incorporating open-ended questions into Genolator’s training set may be the enhancement of its general language understanding. As described above, Genolator was able to clearly distinguish between questions related to different GO aspects [29], a skill it may have developed through exposure to open-ended, generic questions during training. Beyond this aspect-level separation, the inter-GO aspect cosine distance analysis reveals that Genolator's hidden states also capture biologically meaningful relationships across GO aspect boundaries. This suggests that Genolator does not merely learn to separate GO aspects as distinct categories but develops a representation space that encodes the broader biological context and cross-aspect dependencies inherent to the GO hierarchy. While the open-ended responses generated by Genolator are not yet perfect, they demonstrate the potential of this approach to facilitate the exploration and functional annotation of genes that are currently uncharacterized or poorly understood. As the model continues to improve, its ability to generate informative, context-aware answers could provide a valuable resource for hypothesis generation and the investigation of novel gene functions.

### Beyond attention: functional benefits of multimodal token integration in Genolator

To evaluate the contributions of different modalities, we trained two Genolator variants: one incorporating Evo2 and ESM2 embeddings, and another integrating Evo2 and PST embeddings. While on the task of answering confirmative and denial questions both model variants showed a competitive performance, the model receiving the additional information provided by the PST model slightly outperformed the model provided with ESM2 embeddings, as measured by F1-score, MCC (most visible in the questions regarding cellular component) and accuracy. Attention analysis of the Evo2 + ESM2 model revealed that model attention was predominantly focused on Evo2 tokens, with mean attention scores exceeding 0.6 across most transformer layers, while ESM2 embeddings garnered only minimal attention, primarily in the first layer. This pattern was mirrored in the virtual token ablation study: ablating ESM2 tokens led to a modest 0.51% increase in cross-entropy loss for open-ended tasks, whereas ablating Evo2 tokens resulted in a higher 1.32% loss increase. These results highlight the prevailing importance of Evo2 tokens, with ESM2 providing only minor additional value.

In contrast, integrating PST embeddings in the Evo2 + PST model yielded qualitatively different outcomes. Here, clear and distinct attention patterns were observed for PST tokens, particularly in the first transformer layer, as revealed by both mean attention scores and per-head attention analysis (notably, head 15 in layer 0 with attention weight 0.32). The virtual token ablation for this model demonstrated a more substantial effect: ablating PST tokens resulted in a 3.31% increase in loss, notably higher than the 0.56% increase observed when ablating Evo2 tokens. Interestingly, these PST-related attention patterns were largely restricted to the initial layer, while Evo2 tokens continued to dominate attention in deeper layers (peaking at 0.71 in layer 29), along with prompt and response tokens.

Collectively, these attention and ablation studies demonstrate the clear benefit of incorporating protein structural information via structure-informed protein sequence embeddings (PST). While protein sequence embeddings alone (ESM2) appeared to add minimal value beyond DNA embeddings, the inclusion of structure-enriched embeddings (PST) provided a substantial and unique contribution, despite PST having a lower intrinsic dimensionality than ESM2. This indicates that the structural context captured by PST enriches the model beyond what can be achieved with sequence modality alone.

These results further support two important takeaways. First, although attention weights can suggest feature or token importance, they do not always correlate directly with other importance measures (such as ablation impact) and may fail to capture the full picture, especially when examining only mean or last-layer attentions. This observation is consistent with the findings of Jain and Wallace (“Attention is not Explanation”) [55] and Wiegreffe and Pinter (“Attention is not not Explanation”) [56]. As such, a multimodal approach to importance assessment, combining attention analysis and systematic ablation, provides more robust insight into model behaviour. Second, our ablation study also highlights the limitation of cross-entropy loss as a sole qualitative metric: even when the loss increase is limited (e.g., up to 3.72% with both Evo2 and PST tokens ablated), the resulting model answers are practically unusable and completely off, as no genomic context is provided. Since the model continues to generate answers, albeit of poor quality, cross-entropy loss alone may underestimate the true performance impact of losing relevant modality information.

## Conclusion

Genolator is a generative machine learning model, fusing natural language with the language of life, the genetic code. We highlight that Genolator effectively leverages its language processing capabilities together with a comprehensive integration of genomic information, as demonstrated by its strong performance in both the confirmation/denial question dataset and solid capability in open-ended question answering tasks. An analysis of its hidden states reveals that Genolator has a fused understanding of both languages, as visible in the provided t-SNE [44] and cosine similarity plots.

Importantly, this work underscores the technical achievement in multimodal integration and demonstrates new ways to interact with genomic data. As natural language interfaces continue to advance, tools like Genolator pave the way for more intuitive and accessible engagement with complex biological information expanding the possibilities for research, diagnostics, and discovery, particularly regarding poorly characterized protein coding genomic regions. An interesting direction for future work is the integration of Genolator into a multi‑agent system, where it could serve as an intermediary between natural language and genomic models.

While Genolator already demonstrates strong performance, the adoption and training of even larger and more powerful language models might yield further improvements. Continued research will focus on enhancing Genolator’s capacity to answer open-ended questions and broadening its applicability to an ever-wider range of genomic and biomedical tasks.

## Methods

### Construction of custom QA datasets

As a foundation for our QA datasets, we utilized the MANE (Matched Annotation from NCBI and EMBL-EBI [57]) Select release 1.4 (GRCh38), which provides single representative transcripts for each canonical protein-coding gene. For each gene, we retrieved both the DNA sequence using BioPython [58] and the corresponding amino acid sequence. Genes were further annotated with gene terms (GO) [29] encompassing all three aspects: Molecular Function, Cellular Component, and Biological Process.

To ensure the integrity of our fine-tuning and evaluation, we explicitly constrained the data generation process: gene names, gene symbols, the raw DNA sequences, and amino acid sequences were never included/provided in any generated QA samples, prompts, or natural language context. This measure was taken to ensure that the model cannot leverage prior learned knowledge about genes by gene names or recognizable DNA/amino acid sequences to infer the correct response, thereby enforcing that all answers are derived from reasoning over the multimodal integration of provided genomic DNA, amino acid and protein structure embedding.

For each GO aspect [29], we engineered specific system prompts to guide a GPT 4.1 [38] model (hosted on Azure AI Foundry) in generating three confirmation QA samples per gene, leveraging the ontology annotations and at least six manually crafted few-shot examples from two or more unrelated genes per prompt. These samples formed the confirmation QA datasets.

To construct denial QA samples, we applied an analogous strategy: three denial samples were generated per aspect and gene by instructing the GPT 4.1 [38] model to formulate questions regarding associations with GO terms that were not present in the gene´s ground truth annotations. This approach ensured that denial samples specifically addressed molecular functions, biological processes or cellular components not – at least so far – attributed to the target gene.

Furthermore, for each GO aspect [29] and gene, we also engineered a generic QA sample using a dedicated system prompt designed to elicit broad, open-ended questions and answers about potential biological processes, molecular functions or cellular components that could be associated with a given sequence or structure. This prompt excludes mention of specific gene names, protein names, pathway names, mechanisms, or nucleic acid terminology, and focuses instead on plausible broad processes/functions/components, with responses tailored to reflect the main biological process, molecular function and cellular component associated with the GO term [29] provided.

In total, we produced nine datasets:

1. Confirmation QA: biological process
2. Confirmation QA: cellular component
3. Confirmation QA: molecular function
4. Denial QA: biological process
5. Denial QA: cellular component
6. Denial QA: molecular function
7. Generic QA: biological process
8. Generic QA: cellular component
9. Generic QA: molecular function

Each confirmation and denial dataset comprised approximately 54,000 QA pairs (three per ~ 18,000 genes), while each generic dataset consisted of 18,000 QA samples (one per gene), yielding a total of around 378,000 samples across all datasets. To ensure and estimate dataset quality, QA samples from a random selection of 56 genes were independently and blindly reviewed by two researchers, who annotated each sample as “accepted,” “declined,” or “review.” The system prompts and few-shot examples utilized are provided as an Excel sheet in Additional file 2 titled *“Prompts.”*

**Fig. 13:**
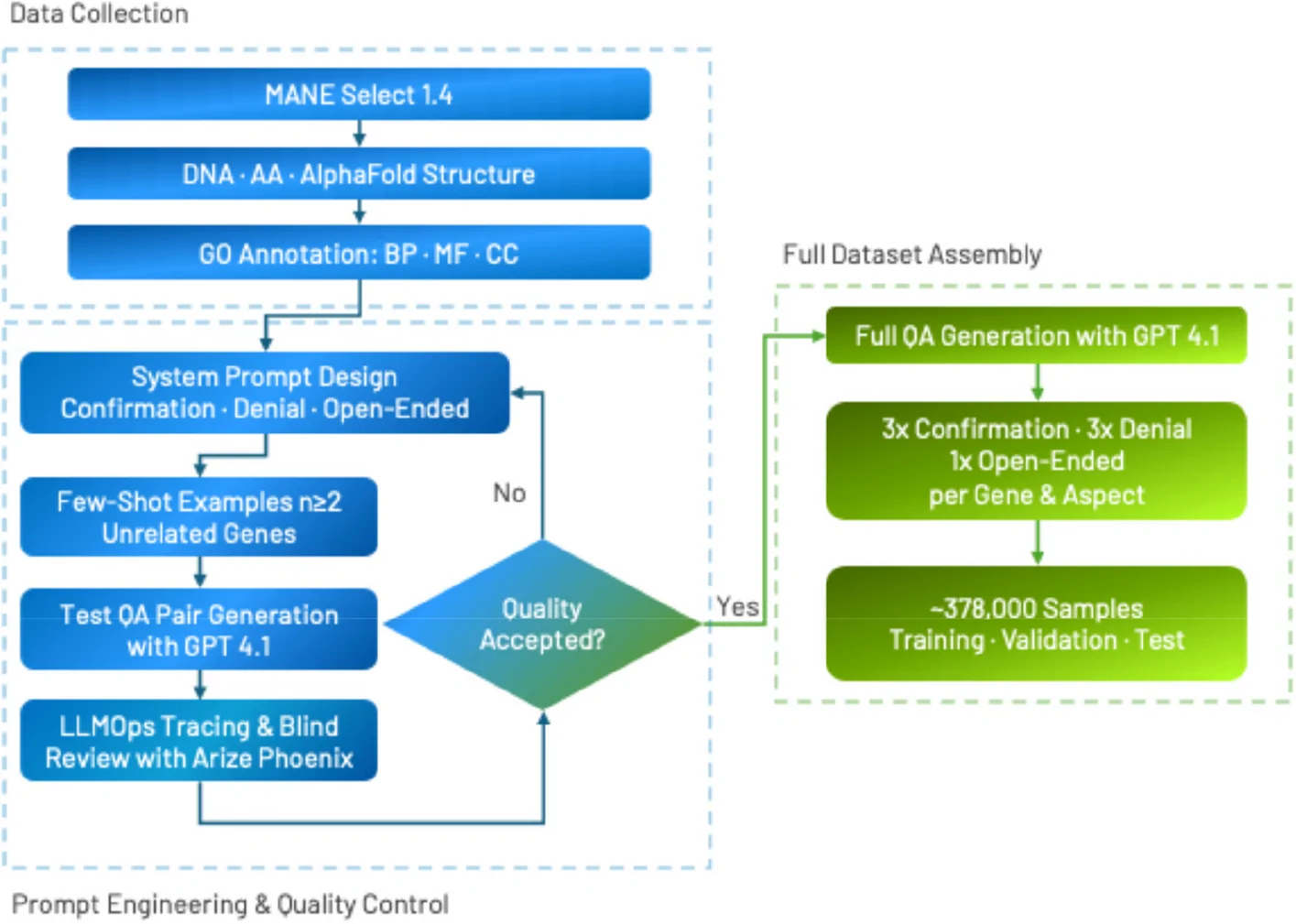
QA Dataset creation workflow

For this and to support LLM evaluation and monitoring, we employed Arize Phoenix, an open-source platform for LLM operations, trace collection, and analytics. A dedicated Phoenix instance was self-hosted on Azure using Azure App Service for deployment, SQLite in combination with Azure Data Lake Gen2. This infrastructure enabled systematic analysis, annotation, and evaluation of the LLM generated QA samples throughout the project lifecycle. Lastly, Protein structural data was obtained from the AlphaFold protein structure database [59–61] in form of PDB-files. The dataset creation workflow is visualised in Fig. 13.

### Genomic sequence embeddings with Evo2

To obtain compact genomic representations, we utilized Evo2 [22] 7B, a large-scale genomic foundation model trained on 9.3 trillion DNA base pairs. For each gene's DNA sequence, per-token embeddings were extracted from the ‘blocks.28.mlp.l3’ layer, following the model authors’ recommendations. These token-level embeddings were then aggregated using mean pooling along the sequence dimension, resulting in a fixed-size 4096-dimensional embedding vector for each gene. Evo2 [22] was deployed and inference was conducted on an Azure Standard_NC40ads_H100_v5 virtual machine, equipped with 40 CPU cores, 320 GB RAM, and a single NVIDIA H100 GPU.

### Protein sequence embeddings with ESM2

Amino acid sequence embeddings were generated using the ESMFold [36] (3B) model accessed via Hugging Face [62] and deployed on an Azure Standard_NC24ads_A100_v4 virtual machine (24 CPU cores, 220 GB RAM, and one NVIDIA A100 GPU). Due to the O(n^3^) complexity of the ESMFold [36] model, longer sequences occasionally led to memory overflows. To address this, protein sequences exceeding 1,000 amino acids were divided into subsequences of 1,000 residues each, with model inference performed separately on each segment.

For each token of the sequence, embeddings from the last hidden layer of the ESM2 block were extracted and concatenated, yielding per-token vectors of length 2,560. These token-level embeddings were aggregated via mean pooling along the sequence dimension, resulting in a single 2,560-dimensional embedding vector per gene.

### Structural enriched protein sequence embeddings with PST

Structural enriched protein (amino acid) sequence embeddings were generated using a Protein structure transformer (PST). The PST model augments the pretrained ESM2 protein sequence embeddings by infusing them with structural knowledge, thereby enabling the extraction of representations that capture both sequence and structural features, as described by Hartout et al. [37]. For each gene, the protein structure of the MANE transcript was retrieved from the AlphaFold protein structure database. The corresponding PDB files were then processed with the PST model, which assigned a 1280-dimensional embedding to each amino acid in the protein sequence. These amino acid embeddings were subsequently aggregated by mean pooling to obtain a fixed-size representation for each protein.

### Multimodal projection into the Llama embedding space and Llama fine-tuning

As a base model we selected Bio-Medical-Llama-3 8B provided by ContactDoctor on Hugging Face, an 8-billion parameter version of Llama-3 that is pre-fine-tuned on biomedical samples for improved natural language understanding in medical and genetics contexts.

To enable multimodal integration within the Llama architecture, we mapped the embeddings representing the DNA sequences (Evo2 [22]), amino acid sequences (ESM2 [36]), or protein structures (PST) into Llama’s native token embedding space. This was achieved by designing three separate virtual token projectors, one per modality, with identical architecture. Each projector applied a learnable linear transformation to map the input embedding dimension to the hidden size expected by Llama (4096). For each sample, eight virtual tokens per modality were produced, yielding a total of 16 multimodal virtual tokens (Evo2 + ESM2 or Evo2 + PST), which were concatenated and prepended to the textual input tokens for downstream model processing. Prior to concatenation, we rescaled the projected Evo2, ESM2 and PST virtual token embeddings to match the average norm of the text token embeddings. For each batch, a scalar text embedding norm $t=mean\Vert {E}_{text}\Vert$ and modality norms $d=mean\left(\Vert {E}_{Evo2}\Vert \right)$, $p=mean\left(\Vert {E}_{PST}\Vert \right)$ or $a=mean\left(\Vert {E}_{ESM2}\Vert \right)$, are computed by averaging over tokens and batch. Scaling factors are then defined as ${\alpha}_{Evo2}=\frac{t}{\left(d+1{e}^{-8}\right)}$, ${\alpha}_{PST}=\frac{t}{\left(p+1{e}^{-8}\right)}$ or ${\alpha}_{ESM}=\frac{t}{\left(a+1{e}^{-8}\right)}$, and the modality embeddings are rescaled as ${E}_{Evo2}\leftarrow {\alpha}_{Evo2}*{E}_{Evo2}$, ${E}_{PST}\leftarrow {\alpha}_{PST}*{E}_{PST}$ or ${E}_{ESM}\leftarrow {\alpha}_{ESM}*{E}_{ESM}$. This normalization aligns the magnitude of multimodal virtual tokens with the language model’s native token space, making the tokens more usable by the Llama model. Without normalization, fine-tuning with modality tokens that dominate the scale of text tokens can result in the suppression of newly introduced virtual tokens, hindering effective multimodal integration.

**Fig. 14:**
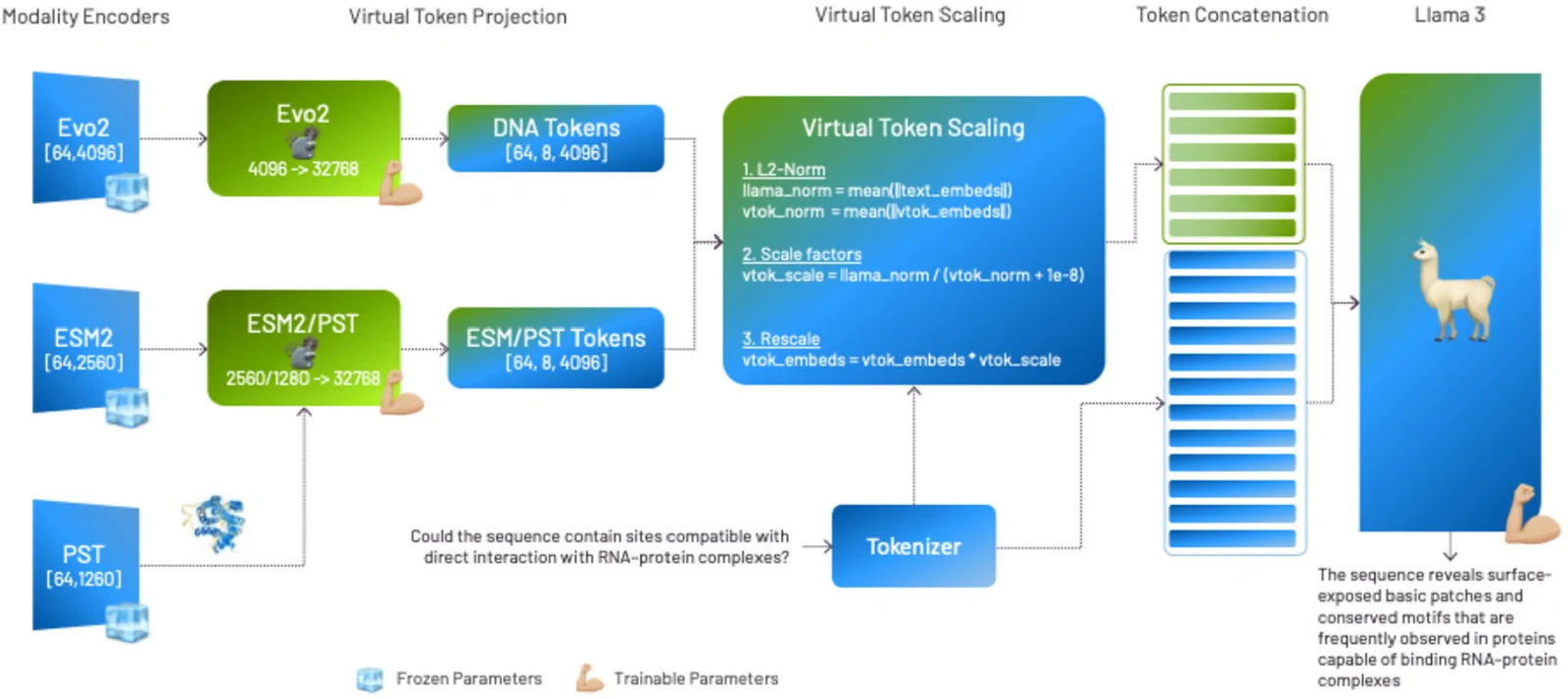
Schematic Illustration of the multimodal fine tuning using feature fusion via token concatenation with tokens derived from genomic as well as protein sequences and protein structures

Followingly, all token projectors, as well as the Llama [33] model itself, were fine-tuned jointly on the QA datasets. To accelerate fine-tuning and efficiently enable parameter-efficient adaptation, we utilized the Unsloth [63] framework in combination with LoRA (Low-Rank Adaptation) [64]. Unsloth facilitated fast model loading and optimized training routines, including support for extended sequence lengths and hardware-aware precision selection. LoRA adapters were applied to all attention projection modules ("q_proj", "v_proj", "k_proj", "o_proj", "gate_proj", "up_proj", "down_proj"). Experiments, metrics, parameters and tokenization details were tracked using Azure Machine Learning and MLflow for experiment reproducibility. During training, the weights of the original foundation models (Evo2, ESM2 and PST) used to generate the modality-specific embeddings) remained frozen, ensuring that only the virtual token projectors and the Llama model were updated. With this design, we trained two distinct models: one utilizing Evo2 and ESM2 virtual tokens for DNA and amino acid sequence embeddings, and another utilizing Evo2 and PST tokens for DNA and protein structure-infused amino acid embeddings. The first model incorporated sequence-level representations from Evo2 (DNA sequence) and ESM2 (amino acid sequence), while the second model integrated Evo2 and PST embeddings, enabling the inclusion of structural enriched amino acid sequence features information alongside DNA in the virtual token embeddings. In both models, two distinct embedding types were provided as input, ensuring comprehensive multimodal representation. Figure 14 provides a visual overview of the proposed multimodal model architecture, including the virtual token projection and integration of DNA, sequence, and structure embeddings within the Llama backbone.

In addition to this joint fine-tuning approach, we also conducted a comparative experimental setup in which the Llama [33] backbone was kept fixed and only the token projectors were trained. This enabled us to assess the impact of updating the core language model versus solely adapting the modality-specific projectors.

### Similarity-aware dataset splitting

While conventional approaches often employ gene-based splits (assigning mutually exclusive gene names to training, validation, and test sets) to prevent direct data leakage and inflated performance estimates, this method has notable limitations. Specifically, partitioning solely by gene names does not fully eliminate the risk of information leakage, as homologous genes or those with highly similar sequence or structural features may still be distributed across multiple sets. Recognizing these shortcomings, we instead adopted a more robust, similarity-aware partitioning strategy, as described below:

To prevent data leakage not just at the gene level but also across related sequences and structures, we adopted a similarity-aware partitioning protocol based on clustering in the multimodal embedding space. For each gene, the DNA, protein, and structure embeddings were concatenated to form a unified multimodal representation. These vectors were normalized and clustered via KMeans [39] (k = 3) using cosine similarity to ensure that structurally or functionally similar genes would be assigned to the same cluster.

Clusters were then mapped to train, validation, and test splits, with cluster sizes adjusted if necessary to achieve an approximate 80%/10%/10% data division. Assignment to each split was further refined using distance to cluster centroids, prioritizing samples most central to each cluster and ensuring exclusivity of samples across splits. This approach reduces the risk of intergenic data leakage, yielding more robust evaluations of model generalizability.

### Fine-tuning and experimental setup

For efficiency, the training split was divided into three further equal-sized subsets. During each epoch, two of these were used, exposing every training point at least once every three epochs. Training was conducted for up to ten epochs, with a batch size of eight, a learning rate of ${5*10}^{-5}$ and employed early stopping (patience = two epochs) based on validation loss. Model validation was performed after each epoch, with final selection and reporting based on results from the strictly held-out test set. The training was conducted using Hugging Face [62], PyTorch [65], Unsloth [63] and MLflow [66] was used for experiment tracking. The training and inference code implementing the procedure described above is available on GitHub and archived on Zenodo [67]. The trained Multimodal-Llama (Evo 2 + PST) variant [68] and the Multimodal-Llama (Evo 2 + ESM-2) variant [69] are deposited on Hugging Face. The multimodal question–answer dataset assembled for this study is deposited on Hugging Face [70].

### Model evaluation

#### Confirmation/denial task (binary classification)

To assess each model’s ability to confirm or deny an association between a given (unknown) sequence and a specified biological function, process, or component, we framed evaluation as a binary classification task. The following six models were compared:

1. Multimodal-Llama (Evo2 + ESM2/Full Fine-Tuning): Our multimodal Llama model in which both the virtual token projectors and the Llama backbone were fine-tuned. In this model, the virtual tokens themselves were based on Evo2 and ESM2 embeddings.
2. Multimodal-Llama (Evo2 + PST/Full Fine-Tuning): Our multimodal Llama model in which both the virtual token projectors and the Llama backbone were fine-tuned. In this model, the virtual tokens themselves were based on Evo2 and PST embeddings.
3. Multimodal-Llama (Evo2 + ESM2/TP-Only Fine-Tuning): An ablated variant where only the token projectors were trainable, and the Llama backbone was held fixed. In this model, the virtual tokens themselves were based on Evo2 and ESM2 embeddings.
4. Multimodal-Llama (Evo2 + PST/TP-Only Fine-Tuning): An ablated variant where only the token projectors were trainable, and the Llama backbone was held fixed. In this model, the virtual tokens themselves were based on Evo2 and PST embeddings.
5. XGBoost Binary Classifier: A non-generative baseline leveraging concatenated prompt (BERT embeddings), and PST embeddings as input features for standard binary classification.
6. GPT 4.1 Prompted Classifier: A system-prompted GPT 4.1 model tasked with direct classification using the DNA and amino acid sequences appended as plain text in the prompt.

### Evaluation protocol

1. Each model was prompted using question–answer test samples from the held-out KMeans split.
2. For generative models, responses were labelled as "confirmation" or "denial" by an automatic GPT 4.1 post-processor.
3. Predictions from all models were compared to ground truth binary labels.
4. Performance was assessed using precision, recall, F1-score, and Matthew’s correlation coefficient (MCC).

#### Reasoning assessment on generic QA (open-ended questions)

To evaluate the capacity for generative reasoning, only the best fine-tuned multimodal Llama model (Evo2 + PST/Full Fine-Tuning) was considered. Here, the model was prompted with open-ended questions regarding the possible biological functions, processes, or components associated with specific sequences.

#### Evaluation approach

**Fig. 15:**
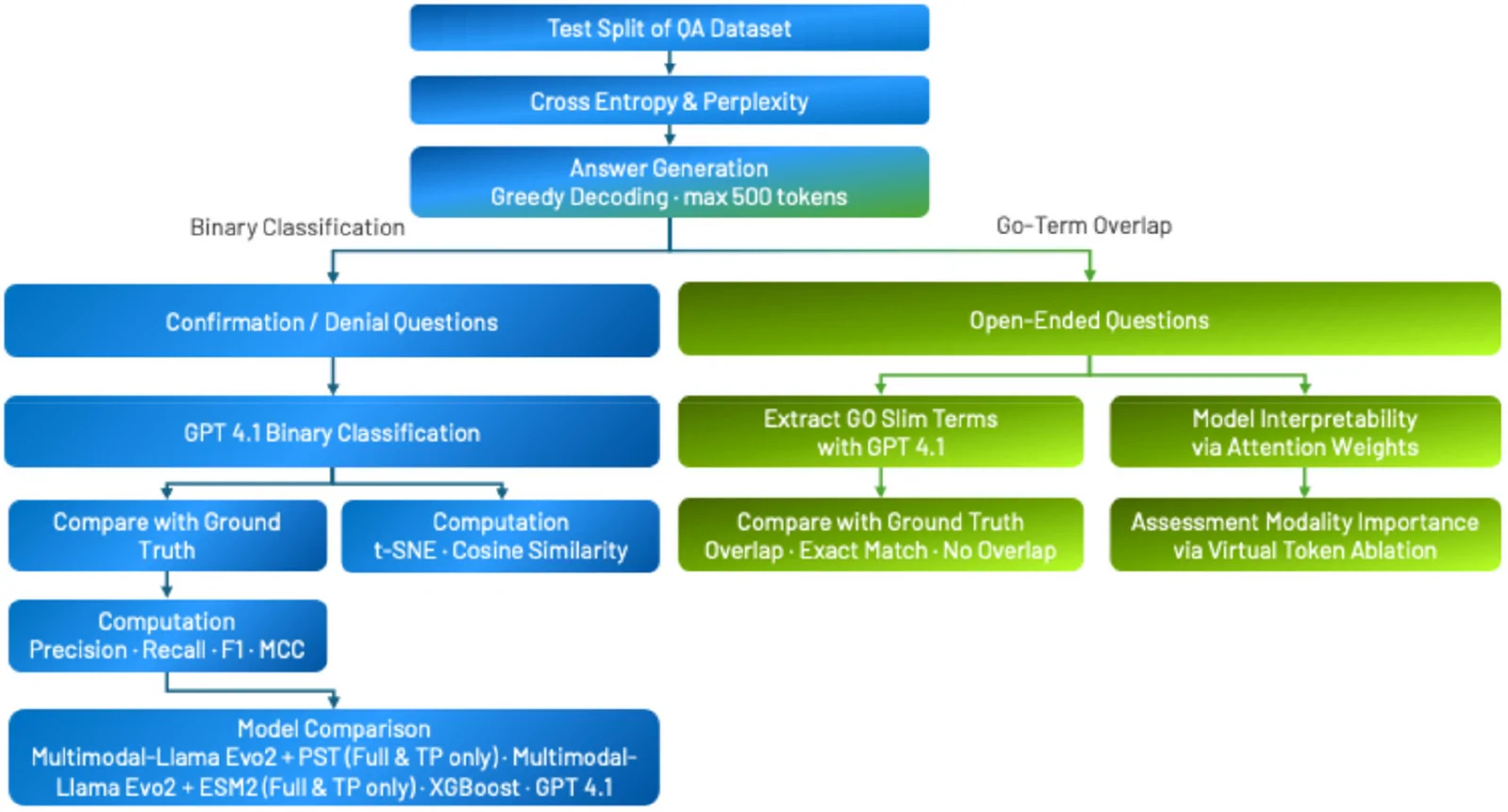
Model Evaluation Workflow using the test split of the QA Dataset

Generated responses were analysed using GPT 4.1 and a Gene Ontology (GO) Slim subset, extracting a simplified set of GO terms from both model and reference answers. The evaluation workflow is visualised in Fig. 15.

Overlap and accuracy were evaluated using:

1. The fraction of samples with overlapping model-generated and reference GO Slim terms.
2. The fraction with exact set matches.
3. The fraction with no overlap.

This two-pronged evaluation strategy provided both quantitative benchmarking of classification performance and qualitative assessment of the generative reasoning capabilities of the multimodal Llama model. The system prompts and few-shot examples utilized are provided as an Excel sheet in Additional file 2 titled *“Prompts.*”

### Model interpretability, representation analysis, and modality assessment

To gain deeper insights into the internal mechanisms and learned representations of our models, we employed two complementary interpretability analyses: visualization of hidden states via t-distributed stochastic neighbour embedding (t-SNE) [44] and pairwise cosine similarity, alongside a fine-grained examination of attention weights and virtual token importance across modalities.

### Feature space visualization with t-SNE

To assess whether the model effectively integrated natural language and biological information, we extracted the hidden representations from the final Llama layer for each input-response pair. The mean or aggregated hidden states for each sequence were projected into two-dimensional space via t-SNE, allowing us to visualize the structure and clustering of learned embeddings. This analysis aimed to reveal whether the model developed a feature space in which natural language and biological contextual information were meaningfully combined, and whether generalizable genomic or functional rules emerged from fine-tuning. 

While t-SNE is a powerful tool for visualizing high-dimensional data, it is primarily intended for exploratory visualization rather than quantitative analysis. Clusters observed in t-SNE plots typically reflect local structure and neighbourhood relationships, but do not necessarily correspond to global similarity or distance between sequences [71, 72]. To address these limitations, we complemented our visualization with a quantitative analysis by computing pairwise cosine similarity between gene ontology (GO) terms within each GO aspect. Specifically, the hidden states associated with each GO term were aggregated by mean pooling, enabling a direct and quantifiable assessment of representation similarity in the model’s native embedding space (4096).

### Model interpretability via attention weights

To gain a deeper understanding of the model’s decision-making processes and the roles of each modality during answer generation, we conducted a comprehensive and fine-grained analysis of attention weights. Beyond simply averaging attention weights from the last hidden layer, we systematically captured attention patterns across all 32 transformer layers and, within each layer, for all 32 attention heads. This approach enabled a detailed assessment of how attention was allocated among virtual token representations (corresponding to DNA, amino acid sequence, and/or structure), the input prompt, and the generated response tokens throughout the generation process. By quantifying modality-specific attention distributions over both layers and heads, we were able to reveal intricate attention patterns and how they evolved temporally and hierarchically across the network. This framework allowed us to characterize not only the integration of multimodal knowledge but also provided insights into the specific contributions of DNA, amino acid sequence, and structural information in guiding the model’s answers.

### Assessment of modality importance via virtual token ablation

While attention weights are widely used as an interpretability tool and offer valuable insights into how models focus on different tokens or modalities; they must be interpreted with caution. As demonstrated by Jain and Wallace (2019), attention weights do not always correlate with other measures of feature importance, such as those obtained through ablation studies. To compensate for these limitations and obtain a more robust estimate of modality importance, we systematically masked the virtual tokens corresponding to individual modalities (e.g., DNA, protein sequence, or structure) during inference by replacing their embeddings with zero vectors and measured the resulting increase in model loss. By comparing the loss incurred when each modality was ablated against the loss observed with all modalities present, we directly quantified each modality’s importance for answer generation [55, 56].

## Supplementary Information

Additional file 1: Table S1 results of the small case study utilizing GPT 4.1. Figures S1-S13: t-SNE visualizations. Figures S14-S16: cosine distance heatmaps. Figures S17-S20: example panels.

Additional file 2.

## Data Availability

The code developed for this study is publicly available on GitHub: https://github.com/IHGGM-Aachen/Genolator, released under the MIT license. The state of the code at the time of publication is additionally archived on Zenodo: https://doi.org/10.5281/zenodo.22008517. The Genolator models are publicly available on Hugging Face: https://doi.org/10.57967/hf/10054 & https://doi.org/10.57967/hf/10055. The datasets created for this study are publicly available on Hugging Face as well: https://doi.org/10.57967/hf/10034.
