# Supplementary material for: Genolator enables protein function interpretation using a multimodal large language model fusing genomic and structural interpretation with natural language interaction
Source: Genome Biol. 2026 Sep 16;27:290. doi: 10.1186/s13059-026-04274-w (PMC13579901; doi:10.1186/s13059-026-04274-w)
Supplement: Supplementary file 1 [file 13059_2026_4274_MOESM1_ESM.docx]

**Genolator enables protein function interpretation using a multimodal large language model fusing genomic and structural interpretation with natural language interaction (supplementary figures and tables)**

Martin Danner^1,2^, Tanhim Islam^1^, Matthias Begemann^1^, Florian Kraft^1^, Miriam Elbracht^1^, Ingo Kurth^1^, Jeremias Krause^1^

^1^Center for Human Genetics and Genomic Medicine, Medical Faculty, Uniklinik RWTH Aachen, Pauwelsstrasse 30, Aachen, 52074, North-Rhine-Westphalia, Germany.

^2^scieneers GmbH, Kantstrasse 1a, Karlsruhe, 76137, Baden-Wuerttemberg, Germany.

Correspondence should be addressed to:

Jeremias Krause

Institute for Human Genetics and Genomic Medicine

Medical Faculty, RWTH Aachen University

Pauwelsstrasse 30

D-52074 Aachen

Tel.:	+49 241 87012

E-Mail: jerkrause@ukaachen.de

Supplementary Tables

Table S1: Results of the small case study of GPT 4.1 capabilities to analyse nucleotide sequences and amino acid sequences for aspects of gene functionality.

| Gene | GO Aspect | Associated GO Terms | Predicted Gene  (Blast Results) |
| --- | --- | --- | --- |
| *BRCA1* | Biological Process | - Structural support - Signalling and receptor activity - Enzymatic catalysis - Transcriptional regulation - Transport across membranes | *TP53* (None) |
| *BRCA1* | Molecular Function | - DNA/RNA binding - Transcription Factor - Protein Interactions - Enzyme (kinase, etc) - Signaling/Regulation | *HTT* (None) |
| *BRCA1* | Cellular Component | - Cytoplasm - Nucleus - Membrane | *TP53* (None) |
| *IHH* | Biological Process | - Structural extracellular protein - Cell adhesion, ECM assembly - Tissue structural integrity | *COL1A1* (None) |
| *IHH* | Molecular Function | - Extracellular matrix proteins - Cell adhesion proteins - Protein–protein interactions - Possible nucleic acid binding - Cell–cell or cell–matrix binding - Low Enzymatic Probability | *COL1A1* (None) |
| *IHH* | Cellular Component | - Secreted - Endoplasmatic Reticulum - Plasma membrane | *MPZ* (None) |
| *INS* | Biological Process | - Regulation of Blood Glucose - Carbohydrate, Lipid, and Protein Metabolism - Carbohydrate metabolism - Lipid metabolism - Protein metabolism - Cell Growth and Differentiation - Anabolic Hormonal Effects - Electrolyte Regulation | *INS* (100) |
| *INS* | Molecular Function | - Hormonal Regulation of Metabolism - Signal Transduction - Precursor/Propeptide Processing - Protein Folding and Disulfide Bond Formation - Intercellular Communication | *INS* (100) |
| *INS* | Cellular Component | - Secreted - Endoplasmatic Reticulum | *INS* (100) |

Supplementary Figures

1. t-SNE visualizations (Confirmation/Denial – Evo2 + PST)

Figure S1: t-SNE visualization of Biological Process Gene Ontology (GO) slim terms (0-12), generated using the last hidden states from the fine-tuned Llama model. Each subplot represents a different GO term, displaying embeddings projected into two dimensions (t-SNE 1 and t-SNE 2 axes). Data points are coloured as follows: green (“Confirmed”) indicates the model associates the corresponding gene with the GO term; burgundy (“Denied”) indicates the model denies the association; blue (“Unclassified”) indicates genes for which no association was queried.

Figure S2: t-SNE visualization of Biological Process GO slim terms (13-24), generated using the last hidden states from the fine-tuned Llama model. Each subplot represents a different GO term, displaying embeddings projected into two dimensions (t-SNE 1 and t-SNE 2 axes). Data points are coloured as follows: green (“Confirmed”) indicates the model associates the corresponding gene with the GO term; burgundy (“Denied”) indicates the model denies the association; blue (“Unclassified”) indicates genes for which no association was queried.

Figure S3: t-SNE visualization of Biological Process GO slim terms (25-36), generated using the last hidden states from the fine-tuned Llama model. Each subplot represents a different GO term, displaying embeddings projected into two dimensions (t-SNE 1 and t-SNE 2 axes). Data points are coloured as follows: green (“Confirmed”) indicates the model associates the corresponding gene with the GO term; burgundy (“Denied”) indicates the model denies the association; blue (“Unclassified”) indicates genes for which no association was queried.

Figure S4: t-SNE visualization of Biological Process Gene Ontology (GO) slim terms (37-48), generated using the last hidden states from the fine-tuned Llama model. Each subplot represents a different GO term, displaying embeddings projected into two dimensions (t-SNE 1 and t-SNE 2 axes). Data points are coloured as follows: green (“Confirmed”) indicates the model associates the corresponding gene with the GO term;burgundy (“Denied”) indicates the model denies the association; blue (“Unclassified”) indicates genes for which no association was queried.

Figure S5: t-SNE visualization of Biological Process GO slim terms (49-60), generated using the last hidden states from the fine-tuned Llama model. Each subplot represents a different GO term, displaying embeddings projected into two dimensions (t-SNE 1 and t-SNE 2 axes). Data points are coloured as follows: green (“Confirmed”) indicates the model associates the corresponding gene with the GO term; burgundy (“Denied”) indicates the model denies the association; blue (“Unclassified”) indicates genes for which no association was queried.

Figure S6: t-SNE visualization of Biological Process GO slim terms (61-71), generated using the last hidden states from the fine-tuned Llama model. Each subplot represents a different GO term, displaying embeddings projected into two dimensions (t-SNE 1 and t-SNE 2 axes). Data points are coloured as follows: green (“Confirmed”) indicates the model associates the corresponding gene with the GO term; burgundy (“Denied”) indicates the model denies the association; blue (“Unclassified”) indicates genes for which no association was queried.

Figure S7: t-SNE visualization of Molecular Function GO slim terms (0-12), generated using the last hidden states from the fine-tuned Llama model. Each subplot represents a different GO term, displaying embeddings projected into two dimensions (t-SNE 1 and t-SNE 2 axes). Data points are coloured as follows: green (“Confirmed”) indicates the model associates the corresponding gene with the GO term; burgundy (“Denied”) indicates the model denies the association; blue (“Unclassified”) indicates genes for which no association was queried.

Figure S8: t-SNE visualization of Molecular Function GO slim terms (13-24), generated using the last hidden states from the fine-tuned Llama model. Each subplot represents a different GO term, displaying embeddings projected into two dimensions (t-SNE 1 and t-SNE 2 axes). Data points are coloured as follows: green (“Confirmed”) indicates the model associates the corresponding gene with the GO term; burgundy (“Denied”) indicates the model denies the association; blue (“Unclassified”) indicates genes for which no association was queried.

Figure S9: t-SNE visualization of Molecular Function GO slim terms (25-36), generated using the last hidden states from the fine-tuned Llama model. Each subplot represents a different GO term, displaying embeddings projected into two dimensions (t-SNE 1 and t-SNE 2 axes). Data points are coloured as follows: green (“Confirmed”) indicates the model associates the corresponding gene with the GO term; burgundy (“Denied”) indicates the model denies the association; blue (“Unclassified”) indicates genes for which no association was queried.

Figure S10: t-SNE visualization of Molecular Function GO slim terms (37-40), generated using the last hidden states from the fine-tuned Llama model. Each subplot represents a different GO term, displaying embeddings projected into two dimensions (t-SNE 1 and t-SNE 2 axes). Data points are coloured as follows: green (“Confirmed”) indicates the model associates the corresponding gene with the GO term; burgundy (“Denied”) indicates the model denies the association; blue (“Unclassified”) indicates genes for which no association was queried.

Figure S11: t-SNE visualization of Cellular Component GO slim terms (0-12), generated using the last hidden states from the fine-tuned Llama model. Each subplot represents a different GO term, displaying embeddings projected into two dimensions (t-SNE 1 and t-SNE 2 axes). Data points are coloured as follows: green (“Confirmed”) indicates the model associates the corresponding gene with the GO term; burgundy (“Denied”) indicates the model denies the association; blue (“Unclassified”) indicates genes for which no association was queried.

Figure S12: t-SNE visualization of Cellular Component GO slim terms (13-24), generated using the last hidden states from the fine-tuned Llama model. Each subplot represents a different GO term, displaying embeddings projected into two dimensions (t-SNE 1 and t-SNE 2 axes). Data points are coloured as follows: green (“Confirmed”) indicates the model associates the corresponding gene with the GO term; burgundy (“Denied”) indicates the model denies the association; blue (“Unclassified”) indicates genes for which no association was queried.

Figure S13: t-SNE visualization of Cellular Component GO slim terms (25-26), generated using the last hidden states from the fine-tuned Llama model. Each subplot represents a different GO term, displaying embeddings projected into two dimensions (t-SNE 1 and t-SNE 2 axes). Data points are coloured as follows: green (“Confirmed”) indicates the model associates the corresponding gene with the GO term; burgundy (“Denied”) indicates the model denies the association; blue (“Unclassified”) indicates genes for which no association was queried.

2. Cosine distance heatmaps (Confirmation/Denial – Evo2 + PST)

Figure S14: Cosine distance heatmap for Biological Process GO slim terms as represented in Llama-derived hidden state embeddings. Pairwise cosine distances were computed between embeddings of gene ontology (GO) cellular component terms based on the hidden states from the Evo2 + PST model. Darker values represent greater similarity (lower cosine distance), while brighter values indicate greater dissimilarity. The heatmap visually highlights patterns of embedding similarity and potential functional relationships across biological processes.

Figure S15: Cosine distance heatmap for Molecular Function GO slim terms as represented in Llama-derived hidden state embeddings. Pairwise cosine distances were computed between embeddings of gene ontology (GO) cellular component terms based on the hidden states from the Evo2 + PST model. Darker values represent greater similarity (lower cosine distance), while brighter values indicate greater dissimilarity. The heatmap visually highlights patterns of embedding similarity and potential functional relationships across molecular functions.

Figure S16: Cosine distance heatmap for Cellular Component GO slim terms as represented in Llama-derived hidden state embeddings. Pairwise cosine distances were computed between embeddings of gene ontology (GO) cellular component terms based on the hidden states from the Evo2 + PST model. Darker values represent greater similarity (lower cosine distance), while brighter values indicate greater dissimilarity. The heatmap visually highlights patterns of embedding similarity and potential functional relationships across cellular components.

3. Example panels (Confirmation/Denial – Evo2 + PST)

Figure S17: t-SNE visualization for a selection of Molecular Function GO slim terms^29^, generated using the last hidden states from the fine-tuned Llama^33^ model. Each subplot represents a different GO term^29^, displaying embeddings projected into two dimensions (t-SNE 1 and t-SNE 2 axes). Data points are coloured as follows: green (“Confirmed”) indicates the model associates the corresponding gene with the GO term^29^; burgundy (“Denied”) indicates the model denies the association; blue (“Unclassified”) indicates genes for which no association was queried.

Figure S18: t-SNE visualization for a selection of Cellular Component GO slim terms^29^, generated using the last hidden states from the fine-tuned Llama^33^ model. Each subplot represents a different GO term^29^, displaying embeddings projected into two dimensions (t-SNE 1 and t-SNE 2 axes). Data points are coloured as follows: green (“Confirmed”) indicates the model associates the corresponding gene with the GO term^29^; burgundy (“Denied”) indicates the model denies the association; blue (“Unclassified”) indicates genes for which no association was queried.

Figure S19: Cosine distance heatmap for a selection of Molecular Function GO slim terms as represented in Llama-derived hidden state embeddings. Pairwise cosine distances were computed between embeddings of gene ontology (GO) cellular component terms based on the hidden states from the Evo2 + PST model. Darker values represent greater similarity (lower cosine distance), while brighter values indicate greater dissimilarity. The heatmap visually highlights patterns of embedding similarity and potential functional relationships across molecular functions.

Figure S20: Cosine distance heatmap for a selection of Cellular Component GO slim terms as represented in Llama-derived hidden state embeddings. Pairwise cosine distances were computed between embeddings of gene ontology (GO) cellular component terms based on the hidden states from the Evo2 + PST model. Darker values represent greater similarity (lower cosine distance), while brighter values indicate greater dissimilarity. The heatmap visually highlights patterns of embedding similarity and potential functional relationships across cellular components.
